# A Missense Mutation in *PPARD* Causes a Major QTL Effect on Ear Size in Pigs

**DOI:** 10.1371/journal.pgen.1002043

**Published:** 2011-05-05

**Authors:** Jun Ren, Yanyu Duan, Ruimin Qiao, Fei Yao, Zhiyan Zhang, Bin Yang, Yuanmei Guo, Shijun Xiao, Rongxin Wei, Zixuan Ouyang, Nengshui Ding, Huashui Ai, Lusheng Huang

**Affiliations:** Key Laboratory for Animal Biotechnology of Jiangxi Province and the Ministry of Agriculture of China, Jiangxi Agricultural University, Nanchang, China; The Wellcome Trust Centre for Human Genetics, University of Oxford, United Kingdom

## Abstract

Chinese Erhualian is the most prolific pig breed in the world. The breed exhibits exceptionally large and floppy ears. To identify genes underlying this typical feature, we previously performed a genome scan in a large scale White Duroc × Erhualian cross and mapped a major QTL for ear size to a 2-cM region on chromosome 7. We herein performed an identical-by-descent analysis that defined the QTL within a 750-kb region. Historically, the large-ear feature has been selected for the ancient sacrificial culture in Erhualian pigs. By using a selective sweep analysis, we then refined the critical region to a 630-kb interval containing 9 annotated genes. Four of the 9 genes are expressed in ear tissues of piglets. Of the 4 genes, *PPARD* stood out as the strongest candidate gene for its established role in skin homeostasis, cartilage development, and fat metabolism. No differential expression of *PPARD* was found in ear tissues at different growth stages between large-eared Erhualian and small-eared Duroc pigs. We further screened coding sequence variants in the *PPARD* gene and identified only one missense mutation (G32E) in a conserved functionally important domain. The protein-altering mutation showed perfect concordance (100%) with the QTL genotypes of all 19 founder animals segregating in the White Duroc × Erhualian cross and occurred at high frequencies exclusively in Chinese large-eared breeds. Moreover, the mutation is of functional significance; it mediates down-regulation of *β-catenin* and its target gene expression that is crucial for fat deposition in skin. Furthermore, the mutation was significantly associated with ear size across the experimental cross and diverse outbred populations. A worldwide survey of haplotype diversity revealed that the mutation event is of Chinese origin, likely after domestication. Taken together, we provide evidence that PPARD G32E is the variation underlying this major QTL.

## Author Summary

A central but challenging objective in current biology is to dissect the genetic basis of quantitative traits. Numerous quantitative trait loci (QTL) have been uncovered in model and farm animals, providing unexpected insights into the biology of complex traits. However, only a few causal variants underlying the QTL have been explicitly identified. By using a battery of genetic and functional assays, we herein show that a major QTL effect on pig ear size is most likely caused by a single base substitution in an evolutionary conserved region of the *PPARD* gene. The protein-altered mutation is of functional significance and explains a proportion of variation in ear size across diverse pig breeds. A worldwide survey showed that the mutant allele for increased ear size was derived from a common ancestor in Chinese pigs, likely after domestication. These findings establish, for the first time, an essential role of PPARD in ear development and highlight the great potential of naturally occurring mutations in farm animals to gain insights into mammalian biology. Moreover, the knowledge of the *PPARD* causal mutation adds to the limited list of quantitative trait genes and quantitative trait nucleotides characterized in domesticated animals.

## Introduction

**Figure 1:**
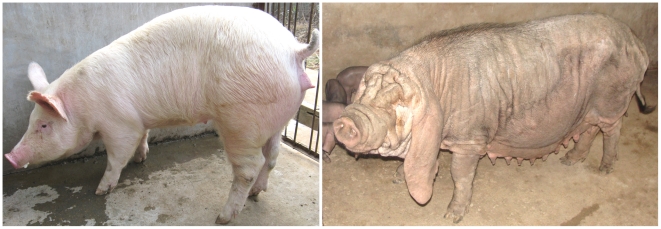
The Erhualian and White Duroc phenotypes. Erhualian pigs (right panel) are obese and short legged, have wrinkly face, extremely large and floppy ears. In comparison, White Duroc pigs (left panel) are renowned for muscularity and exhibit much smaller and half or fully pricked ears.

The external ear is part of the auditory system and plays a vital role in collecting sound as the first step in hearing. Multiple congenital anomalies have been documented for human external ears. For instance, microtia, characterized by a small and abnormally shaped outer ear, occurs in approximately one in 8,000–10,000 births. However, only in a minority of cases has a genetic or environmental cause been found [1]. The domestic pig services as not only an agriculturally important animal for meat production but also an important large-animal model for human medicine [2]. Thousands of years of selective breeding has created diversity of phenotypes in pigs, such as ear size in Erhualian and White Duroc breeds. Erhualian is the most prolific pig breed and exhibits unusually large and floppy ears as breed character (Figure 1). Historically, the large-ear feature of Erhualian pigs had been favored by owners for the traditional sacrificial culture [3]. White Duroc is one of worldwide-popular boar line and has small and erect ears (Figure 1). We have created a four-generation White Duroc × Erhualian resource population, in which phenotypic traits related to ear size have been recorded in 1,027 adult F_2_ animals and 560 adult F_3_ individuals (Table S1). We mapped a major QTL for ear size around 58 cM on SSC7 (Figure S1) using a genome scan on the White Duroc × Erhualian cross [4], which confirmed the previously reported QTL affecting ear size in a Large White × Meishan F_2_ resource population [5]. The significant QTL had a small confidence interval of 2 cM and explained more than 40% of phenotypic variance. The aim of this study was to identify the genetic determinant underlying this major QTL.

## Results/Discussion

### Identical-by-descent analysis defines the major QTL within a 750-kb interval

**Figure 2:**
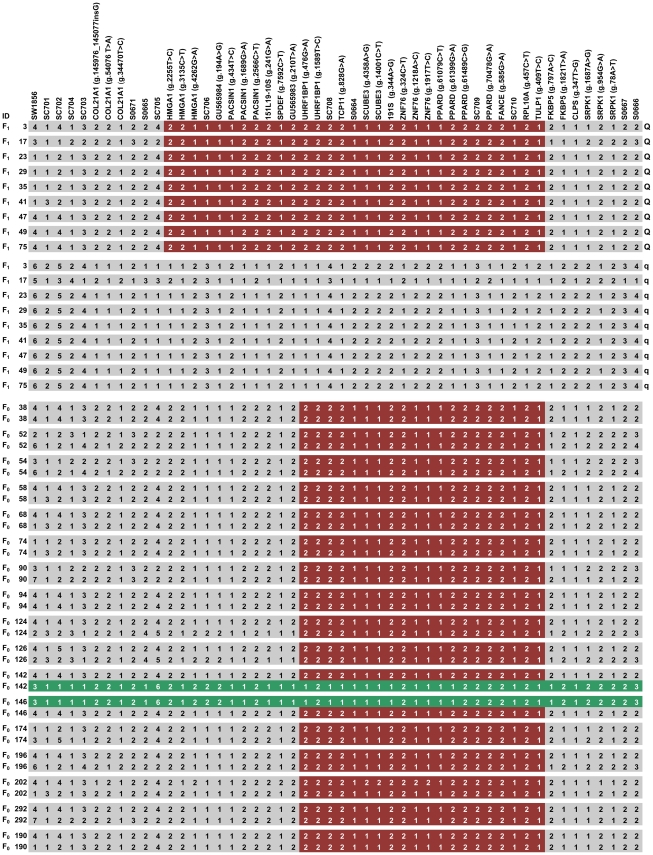
Fine mapping of the QTL by the haplotype sharing analysis. Shared haplotypes of *Q*-bearing chromosomes in F_1_ sires segregating for the QTL and of Erhualian founder chromosomes in the QTL region. Polymorphisms are displayed at the respective gene or microsatellite markers. SNP positions in each gene are given in brackets with reference to GenBank accession numbers. Microsatellite alleles are numbered consecutively from shortest to longest fragments. For SNP markers the allele with the higher frequency is denoted 1, and the allele with the lower frequency is denoted 2. Identities of F_1_ sires and F_0_ Erhualian sows are given in the left axis. QTL genotype of each chromosome of F_1_ sires is shown on the right axis. The shared haplotype blocks are indicated in colored boxes. Two Erhualian founder chromosomes associated with decreased ear size (*E^q^*) are marked in green.

To fine map the QTL, we genotyped 1,027 adult F_2_ animals and their 68 parents and 19 grandparents in the White Duroc × Erhualian cross using additional 17 SNP markers and 11 microsatellite markers in the QTL region. A final set of 33 markers covering the QTL region were then explored to deduce the QTL genotypes of F_1_ sires by the marker-assisted segregation analysis as proposed previously [6]. We determined QTL genotypes of all 9 F_1_ sires (Figure S2). All 9 *Q*-bearing chromosomes for increased ear size shared a haplotype of ∼1.2 Mb flanked by markers *HMGA1* – *TULP1*. The shared haplotype was distinct from *q*-bearing chromosomes (Figure 2). These observations strongly suggest that the QTL is located in the 1.2-Mb interval.

Given the extremely divergent ear size phenotypes between Erhualian and White Duroc animals, we assumed that *Q* and *q* alleles were alternatively fixed in Erhualian and White Duroc founder animals; hence all Erhualian founder sows could share a chromosomal segment carrying the *Q* allele for increased ear size. To test this assumption, we reconstructed haplotypes of all 19 founder animals (2 sires and 17 dams) using 50 markers (15 microsatellites and 35 SNPs) in the QTL region. Almost all Erhualian founder sows shared a haplotype of ∼750 kb within the refined 1.2-Mb interval (Figure 2). As predicted, this shared haplotype was associated with increased ear size and presumably *Q*-bearing chromosomes. Two Erhualian founder sows carried a distinct haplotype (denoted as E^q^), which was unexpected because it was contrast with our initial assumption. We then conducted a statistical analysis of F_2_ animals in the White Duroc × Erhualian cross. The results revealed that the E^q^ chromosome had an effect on decreased ear size similar to the White Duroc chromosome (D^q^) and significantly different from the Erhualian *Q*-bearing chromosome (E^Q^). The least-squares means (± s.e.) of ear weight were 323.07±4.55 for *E^Q^E^Q^* and 266.66±18.9 for *E^Q^E^q^* (*P* = 0.04); 264.71±3.52 for *D^q^E^Q^* and 236.98±17.12 for *D^q^E^q^* (*P* = 0.06, Table 1). The shared E^Q^ chromosome allowed us to refine the location of the major QTL to the 750-kb interval between markers *UHRF1BP1* and *TULP1* (Figure 2).

**Table 1:** Effects of Erhualian *Q* or *q* -bearing chromosomes on ear weight in the White Duroc × Erhualian F_2_ cross.^a^

Genotype	Number	Least square mean ± standard error (g)
D^q^D^q^	197	185.30±4.49^ A^
D^q^E^Q^	443	264.71±3.52^ B^
D^q^E^q^	10	236.98±17.12^ AB^
E^Q^E^Q^	194	323.07±4.55^ C^
E^Q^E^q^	7	266.66±18.9^ B^

**a** E^Q^ represented the major chromosome and E^q^ indicated the other distinct chromosome in Erhualian founder sows. Phenotypic values were corrected for fixed effects including sex, batch and SSC5 QTL for ear size and a covariate of carcass weight. Significance was evaluated by the *t*-test in the GLM procedure of SAS 9.0 (SAS Institute, Cary, NC). Values with different superscripts are significantly different (*P*<0.05).

### Selective sweep analysis refines the QTL to a 630-kb region

**Figure 3:**
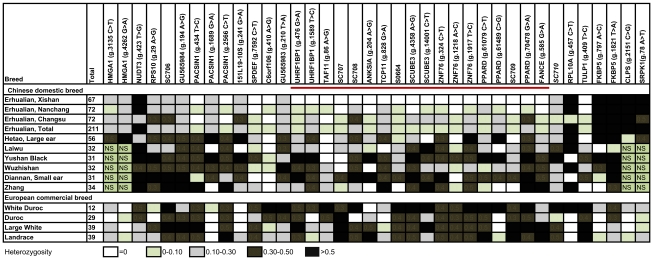
Refining the QTL region by the genetic variation analysis. Heterozygosities of 38 markers of the QTL region in 13 breeds are shown. Numbers of samples in tested breeds are given in parentheses. A near-fixation of alleles (‘selective sweep’) occurs in a 630-kb region between markers *UHRF1BP1* and *FANCE* in Erhualian populations. Sequences corresponding to markers in this figure have been submitted to GenBank with accession numbers GU565968 - GU565989, GU592173.

Historically, Erhualian pigs had undergone selection for ear size because pigs with extraordinary large and floppy ears were favored for the ancient sacrificial culture in the Taihu region of East China [3]. Reduced genetic variation in the critical region containing the QTL was therefore predicted. To define the region of reduced genetic variation, we collected 211 animals representing all lineages in 3 Erhualian nucleus populations, 216 animals from 6 Chinese indigenous breeds and 119 independent animals from 3 Western worldwide-popular commercial breeds. Using these samples, we genotyped 6 microsatellite and 32 SNP markers in the 750-kb region. We found that 18 adjacent markers in a 630-kb region between markers *UHRF1BP1* and *FANCE* showed dramatically reduced polymorphisms in all Erhualian pigs with nearly all major allele frequencies of more than 0.90. Notably, the 18 markers in the 630-kb region are monomorphic in the Erhualian nucleus population from Xishan county (n = 72). In comparison, the genetic polymorphisms of these markers were maintained in other Chinese, Western breeds, and wild boars (Figure 3). The 630-kb region showing strong selective-sweep effects on Erhualian pigs was therefore predicted to contain the responsible locus. We further genotyped the 18 markers in the 630-kb region on 188 adult animals of Sutai pigs. This breed was developed after 18-genereation selection from a Duroc (50%) × Erhualian (50%) cross in 1986 [7], meaning that the breed has undergone 18 generations of meiosis reducing the extent of linkage disequilibrium between QTL and linked markers. The Erhualian-originated haplotype of 630 kb showed significant (*P* = 0.009) association with increased ear size compared with other chromosomes in Sutai pigs (Figure S3), thereby supporting the conclusion that this region harbors the causative gene.

### Positional candidate gene analysis: discovery of a nonconservative missense mutation in *PPARD* concordant with the QTL genotypes of founder animals

The 630-kb region encompasses 9 annotated genes (*ANKS1A*, *DEF6*, *FANCE*, *PPARD*, *SCUBE3*, *TAF11*, *TCP11*, *UHR1BP1* and *ZNF76*) in the human homologous region. RT-PCR was performed to detect expression levels of these genes in ear tissues of piglets. Four genes including *PPARD*, *FANCE*, *TAF11* and *ZNF76* were highly expressed, whereas transcripts of other genes were almost absent in ear tissues (data not shown). Of the 4 genes, PPARD (peroxisome proliferator-activated receptor delta) is a ligand-modulated transcription factor belonging to the nuclear receptor superfamily and plays crucial roles in diverse biologically important processes [8]. For instance, PPARD play a pivotal role in modulating cell differentiation in both keratinocytes and sebocyte of skin [9]. PPARD also serves as a key regulator in fat metabolism; it triggers fat burning and enhances energy uncoupling in adipose tissues and skeletal muscle [10]–[12]. Moreover, PPARD is a key player in Wnt/β-catenin pathway [13], which has essential roles in diverse cellular activities including chondrocyte proliferation and differentiation [14]. The external ear is composed of skin, cartilage, connective tissues and fat. Given its crucial role in skin homeostasis, cartilage development and fat metabolism, *PPARD* stood out as a prime positional candidate for the major QTL. We monitored the relative mRNA expression of *PPARD* in ear tissues of Erhualian and Duroc pigs at four different ages by real-time RT-PCR. The expression levels were higher in samples at early ages (days 0, 45 and 90) compared with adult samples (day 300). However, no significant difference of expression levels was found in ear tissues between large-eared Erhualian and small-eared Duroc pigs (Figure S4).

**Figure 4:**
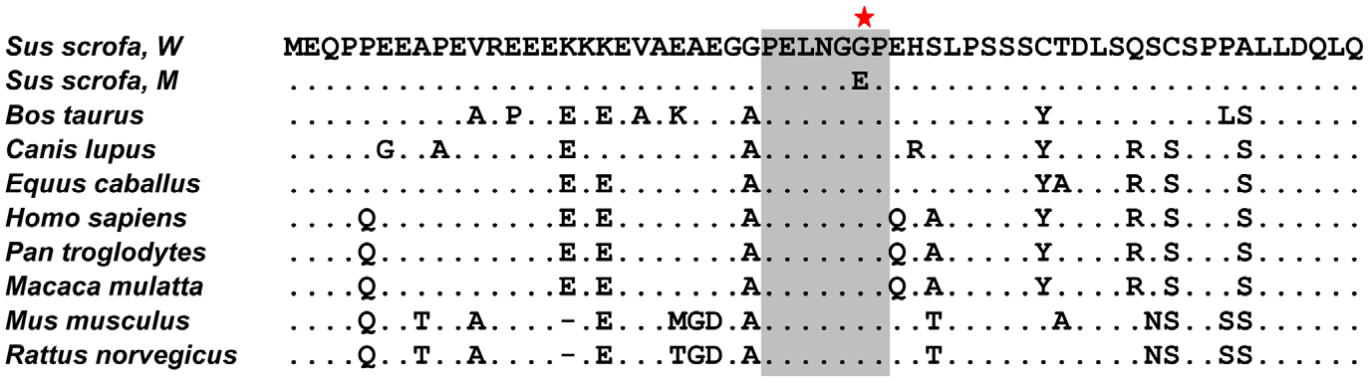
Conservation of the intrinsically disorder domain of PPRAD protein in mammals. The ClustalW alignment of predicted amino acids of 8 orthologous *PPARD* genes is shown. The sequences for the alignment were taken from the following accessions: NP_001123713 and ADF55028 (*Sus scrofa*), NP_001077105 (*Bos taurus*), NP_001041567 (*Canis lupus*), XP_001498920 (*Equus caballus*), NP_006229 (*Homo sapiens*), XP_001172224 (*Pan troglodytes*), NP_035275 (*Mus musculus*) and NP_037273 (*Rattus norvegicus*). The G32E substitution in a conserved hepta-amino acid region (grey box) is indicated by the asterisk. Glycine is the conserved amino acid at this position in the wild-type pigs (*Sus scrofa*, W) and other mammals, whereas glutamic acid occurs only in alleles increasing ear size in pigs (*Sus scrofa*, M).

To search for causative mutations, we first sequenced the entire coding region of the *PPARD* gene using ear mRNA of two White Duroc and two Erhualian animals and identified only one nonsynonymous mutation. The G to A mutation caused a glycine to glutamic acid substitution at codon 32 (GU565977) in the conserved intrinsically disordered domain of the PPARD protein predicted by SMART (http://smart.embl-heidelberg.de/). The intrinsically disordered domain is a distinctive and common characteristic of eukaryotic hub proteins like multifunctional nuclear receptors and serves as a determinant of protein interactivity [15]. Comparison of amino acids of this protein domain across mammals revealed that glycine is well conserved in mammalian PPARDs (Figure 4), while the derived glutamic acid occurs only in alleles increasing ear size in pigs. We thus speculated that the nonconservative substitution probably changes the PPARD interactivity with other protein partners and consequently affects the gene's regulation function. Genotypes of F_1_ sires (9 heterozygotes) and F_0_ animals (17 homozygotes and 2 heterozygotes) at the mutation site were 100% concordance with their QTL genotypes. The potentially altered function and QTL concordance of *PPARD* G32E corresponded to the hypothesis that this SNP may be the causative mutation underlying the major QTL.

### *PPARD* G32E is a functional variant mediating down-regulation of *β-catenin* and its target gene expression

**Figure 5:**
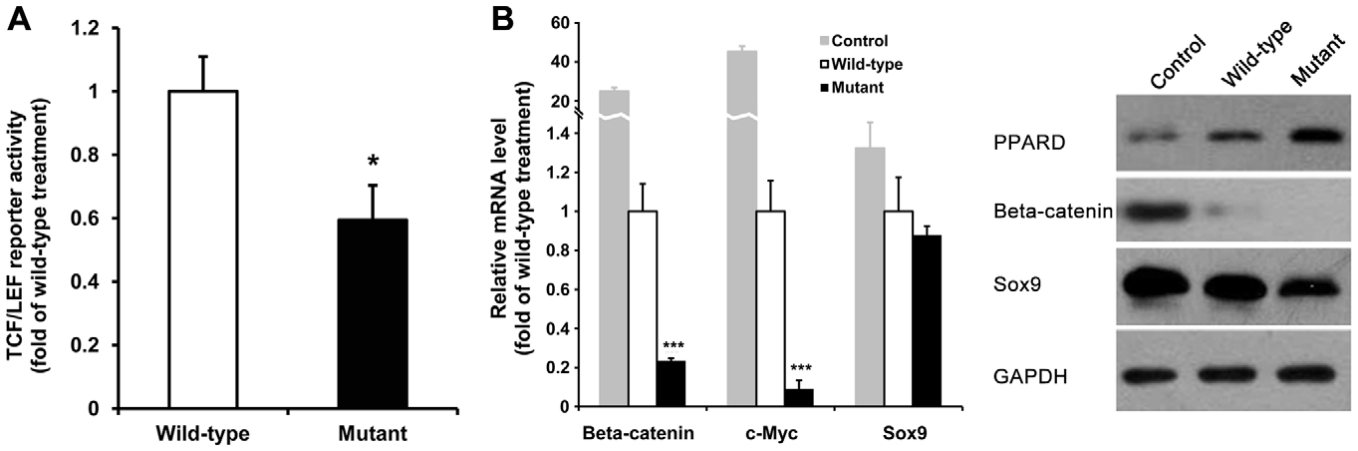
*PPARD* G32E mediates down-regulation of critical genes in the Wnt/*β-catenin* signaling pathway. (A) Overexpression of mutant PPARD decreases TCF/LEF reporter activity. After infection with PPARD lentiviral expression vectors, the 293T cells with 80% confluence were transiently cotransfected with TCF/LEF-Luc reporter vector and a control *Renilla* luciferase expression vector. The normalized luciferase activity was determined as described in Methods. All values are expressed as fold induction relative to basal activity. The figure shown is representative of 3 independent experiments. WT: wild type; Mutant: mutant type. *, *P*<0.05 compared with wild-type treatment. (B) Overexpression of mutant PPARD leads to down-regulation of *β-catenin* and its target gene expression. The pig ear fibroblast cells were transfected with PPARD lentiviral expression vectors for 5 days. Cells were harvested for RNA and protein isolation to assess the mRNA (left panel) and protein (right panel) levels of *β-catenin* and its downstream (*c-myc*) and upstream (*Sox9*) genes by real time quantitative PCR and Western blot assay, respectively. *GAPDH* was used as an internal control. Each value represents the mean ± S.D. of triplicate assays per condition. ***, *P*<0.001 compared with wild-type treatment. Western blot analysis of c-myc protein was failed probably due to the insufficient specificity of rabbit anti-c-myc antibody.

PPARD is involved in the Wnt/β-catenin signaling pathway that regulates diverse cellular functions. In the nucleus, PPARD interacts with β-catenin binding to TCF/LEF transcription factors that stimulate transcription of target genes important for multiple cellular activities including cartilage development and organogenesis [14]. To demonstrate functional significance of *PPARD* G32E, we cotransfected the 293T cells with the lentiviral expression vectors of wild-type or mutant PPARD and a TCF/LEF-driven luciferase reporter construct. A *Renilla* luciferase expression vector was used for the normalization of transfection efficiency. Overexpression of mutant PPARD led to a 40% decrease (*P*<0.05 compared with the wild-type treatment) in TCF/LEF reporter activity (Figure 5A), indicating the G32E mutation mediates down-regulation of *β-catenin* downstream genes. To examine a direct functional role of *PPARD* G32E in target genes of *β-catenin*, we treated pig ear-derived primary fibroblast cells with the lentiviral PPRAD expression vectors and monitored the mRNA levels of *β-catenin* and its known downstream (*c-myc*) [16] and upstream (*Sox9*) [17] genes along with *GAPDH* as a loading control by real time quantitative RT-PCR. The mRNA levels of *β-catenin* and *c-myc* were reduced respectively by 4.1-fold and 11.5-fold (*P*<0.001) in mutant PPARD transfectants compared with the cells transfected with wild-type PPARD. Western blot analysis showed that both β-catenin and c-myc protein levels were decreased by the mutant PPARD treatment (Figure 5B), thereby confirming the results of mRNA and luciferase reporter analyses. *Sox9* mRNA expression in mutant PPARD transfectants was only slightly decreased to 1.1-fold of the wild-type PPARD treatment; the result was validated by Western blot (Figure 5B). GAPDH was used as a protein loading control for total cell lysate, which was not affected by both wild-type and mutant PPARD treatments (Figure 5B). Altogether, we conclude that *PPARD* G32E is a functional variant that mediate down-regulation of *β-catenin* and its target gene expression in the Wnt/β-catenin signaling pathway. Wnt/β-catenin signaling has been firmly demonstrated to suppress adipogensis [18]–[19]. The fact that PPARD is a key modulator of lipid production in the skin [9] and that *PPARD* G32E inhibits *β-catenin* expression led us to assume that the mutation stimulates lipid production and storage that are required for enlarged ear size.

### *PPARD* G32E is significantly associated with ear size across the experimental intercross and outbred populations

To confirm the effect of *PPARD* G32E on ear size, we performed a standard association test, a marker-assisted association test and an *F*-drop test [20] in the White Duroc × Erhualian cross. The SNP showed greatly significant (*P*<0.0001) association with ear weight and ear size in the standard association test. In the marker-assisted association test, the SNP was more significant (*P*<0.001) for these traits compared with the QTL effect. After fitting this polymorphism in the QTL model, the great QTL effect disappeared with *F*-value drop rations of less than 0.03 (Table S2). These results were in agreement with the hypothesis that the SNP is the causative mutation for the major QTL affecting ear size. Nevertheless, we cautioned the results because variants closely linked with a causative mutation also lead to strong association in F_2_ resource populations due to the high level of linkage disequilibrium between founder breeds [20].

To obtain additional supporting evidence, we further genotyped the G32E mutation on 667 mature pigs from 4 Chinese local breeds (Erhualian, Hang, Yushan Black and Bama Xiang) and 3 synthetic commercial lines (Sutai, Suzhong, Sujiang) with phenotypic data of ear size. These populations show a wide range of ear size and segregate for the mutation. The association analyses confirmed the effect of *PPARD* G32E on ear size. The *32E* allele was significantly associated with increased ear size across the tested breeds (*P*<0.05; Table 2). Chinese local pig breeds have low levels of linkage disequilibrium extending up to only 0.05 cM [21]. The concordantly significant association across Chinese breeds thereby strengthened the hypothesis that *PPARD* G32E is the responsible locus for ear size. The effects of *PPARD* G32E differ in their magnitude in the tested breeds; one reason is that the effects are context-dependent and are influenced by different genetic backgrounds and environments. Another possibility is that *PPARD* G32E is only responsible for part of the effect on ear size in Erhualian pigs.

**Table 2:** Effect of the *PPARD* G32E substitution on ear size in 7 outbred populations.

Population	No.	Genotype			*P* value
		GG	GE	EE	
Erhualian	105	-	397.85±15.87 (n = 32)	460.17±10.51 (n = 73)	0.0014
Hang	58	169.01±11.51 (n = 7)	213.4 9± 5.88 (n = 23)	225.38±5.42 (n = 28)	0.0001
Sujiang	80	258.31±5.25 (n = 63)	282.58±10.19 (n = 17)	-	0.0374
Sutai	177	257.29±6.98 (n = 42)	274.62±5.19 (n = 76)	290.45±6.98 (n = 59)	0.0017
Suzhong	81	214.97±6.11 (n = 56)	238.80±9.14 (n = 25)	-	0.0332
Yushan Black	64	-	172.80±5.86 (n = 23)	200.78±4.33 (n = 41)	0.0002
Bama Xiang	102	55.70±1.28 (n = 59)	61.15±1.73 (n = 32)	65.41±2.96 (n = 11)	0.0027

**a** Least square mean ± standard error (cm^2^) is given for each genotype. Significance was evaluated by the GLM procedure of SAS 9.0 (SAS Institute, Cary, NC).

### *PPARD* G32E has a unique origin of Chinese pigs likely after domestication

To reveal the ancestral state and allele frequency of *PPARD* G32E in diverse pig breeds, we genotyped the mutation in a panel of 1,166 animals representing 31 domestic breeds and Chinese and European wild boars. Overall, the derived *32E* allele for increased ear size occurred at high frequencies (>0.80) in Chinese breeds with large and floppy ears. In contrast, the *32G* allele for normal ear size was fixed in all wild boars, European local and commercial breeds, and occurred at low frequencies (<0.30) in Chinese indigenous breeds having small and erect ears. These results indicated that *PPARD* G32E may occur in Chinese pigs after domestication. We detected only one heterozygote in European local breeds (Table 3). The animal was from Large Black pigs that exhibit large and floppy ears and have been influenced by Chinese breeds brought into England in the late 1800′s [22].

**Table 3:** Frequencies of the derived *32E* allele in different ear-sized outbred pig populations.

Phenotype	Breed	Number	Allele frequency
Chinese breeds			
Large and floppy ears	Erhualian, Xishan	67	1.00
	Erhualian, Nanchang	67	0.95
	Erhualian, Wujin	105	0.85
	Erhualian, Changshu	72	0.85
	Hetao Large-ear	55	0.81
	Jiaxing Black	32	1.00
	Meishan	23	0.82
Medium-size and floppy ears	Hang	58	0.68
	Jiangquhai	30	0.07
	Jinhua	30	0.00
	Laiwu	29	0.00
	Lantang	30	0.83
	Minzhu	30	0.67
	Ningxiang	22	0.14
	Rongchang	29	0.38
	Tongcheng	29	0.45
	Yushan Black	64	0.82
Small, erect or half-flicked ears			
	Bama Xiang	32	0.30
	Diannan Small-Ear	31	0.00
	Tibetan	34	0.00
Chinese wild boar		22	0.00
Western breeds			
	Duroc	58	0.00
	European domestic pigs a	28	0.02
	Landrace	71	0.00
	Large White	93	0.00
	White Duroc	12	0.00
European wild boar		13	0.00

**a** European domestic pigs include Iberian (n = 10), Berkshire (n = 4), Large Black (n = 2), Mid White (n = 2), Chester White (n = 2), Old Spot (n = 2), Yorkshire (n = 2), Tamworth (n = 1), British Lop (n = 1), Hampshire (n = 1), Saddle Back (n = 1). All these animals are homozygous *GG* except a heterozygote detected in one of two Large Black pigs, which exhibits large or medium-size and floppy ears and are influenced by Chinese breeds brought into England in the late 1800's (Kijas *et al*. 1998) [22].

We further analyzed the genetic variability and haplotype structure around the G32E mutation in a worldwide pig panel. A total of 868 animals representing 34 breeds were genotyped for 32 SNPs in a 77-kb region of *PPARD*. Again, the Erhualian breed showed a selective sweep signal as it had negative classical selection statistics Tajimas *D* and much smaller nucleotide variability (π_N_) compared with other Chinese local breeds and Western commercial breeds (Table S3). Especially, the genetic variability at the 32 loci was wiped out in the Erhualian population from Xishan. Moreover, we plotted a distribution of the frequency of the derived *32E* allele (*P*_A_) against Tajimas *D* index to elucidate the existence of directional selection for the G32E mutation. When *P*_A_ = 0, Tajimas *D* was highly variable across breeds, likely due to demographic and/or sampling effects. In stark contrast, Tajima's *D* took highly negative values when *P*_A_ >0.8 in Erhualian and other Chinese large-eared breeds as expected in a classical directional selection (Figure S5). We reconstructed 16 major haplotypes with frequencies larger than 0.01 from the 32 SNPs genotyped. Of the 16 haplotypes, only one carried the derived 32E allele; it was at high frequencies in Erhualian pigs and intermediate frequencies in some floppy-eared Chinese breeds whereas absent in Western pigs and wild boars (Table 4). The NJ phylogenetic tree illustrated that the typical haplotype of Erhualian pigs was generally divergent from other haplotypes (Figure S6). These observations supported the assumption that the G32E mutation has a unique origin in Chinese breeds likely after domestication and has undergone selection in Erhualian pigs. We calculated linkage disequilibrium measures (*r* ^2^) between all pairs of loci and inferred haplotype blocks. Three and two haplotype blocks were identified in the *PPARD* region for Chinese indigenous pigs and Western commercial breeds, respectively. Only a single nevertheless larger block that spanned 53 kb and contained the G32E SNP was found in Erhualian pigs, reflecting a selection hitching effect (Figure S7). The G32E SNP was in high disequilibrium with very few of the SNPs analyzed (two with *r^2^*>0.8), and there was no observable trend between physical distance and disequilibrium measures for the G32E SNP and the rest of loci (Figure S8).

**Table 4:** Distribution of major haplotype frequency in the *PPARD* gene in corresponding pig populations.

No. a	Haplotype	All	Erhualian	Chinese local breed	Chinese wild boar	Commercial breed	EU local breed	EU wild boar
Haplo1	CGTGGCGACCATAGTGAGGCTC**A**CTCTCAG	0.42	0.91	0.55	0.00	0.00	0.00	0.00
Haplo2	.AC..TAGT..A.CC.GA.ACT**G**GCGCGGT	0.07	0.01	0.02	0.00	0.26	0.36	0.55
Haplo3	TAC..TAGT..A.CC.GA.ACT**G**GCGCGGT	0.07	0.00	0.02	0.00	0.28	0.15	0.35
Haplo4	......................**G**G....G.	0.06	0.00	0.07	0.17	0.01	0.06	0.05
Haplo5	...A...G...A..CA......**G**G.G..GT	0.06	0.01	0.03	0.02	0.14	0.20	0.00
Haplo6	......................**G**G......	0.04	0.00	0.03	0.00	0.08	0.06	0.00
Haplo7	.......G.TGAG.CA..A...**G**G.G..GT	0.04	0.00	0.04	0.14	0.00	0.04	0.00
Haplo8	......................**G**G.G..GT	0.03	0.00	0.02	0.02	0.08	0.09	0.00
Haplo9	.......G.TGA..CA......**G**G.G..G.	0.03	0.00	0.03	0.14	0.00	0.00	0.00
Haplo10	.......G.TGAG.CA......**G**G.G..GT	0.03	0.00	0.03	0.00	0.00	0.00	0.00
Haplo11	....T..G.TGAG.CA..A...**G**G.G..GT	0.03	0.02	0.03	0.00	0.00	0.00	0.00
Haplo12	TAC..TAGT....CC.GA.ACT**G**GCGCGGT	0.02	0.01	0.02	0.00	0.05	0.02	0.00
Haplo13	..........GAG.CA..A...**G**G.G..GT	0.02	0.00	0.01	0.00	0.06	0.00	0.00
Haplo14	.......G.TGA..CA......**G**G.G..GT	0.02	0.00	0.02	0.10	0.00	0.00	0.00
Haplo15	..C....G.TGA..CA......**G**G.G..G.	0.01	0.00	0.01	0.17	0.00	0.00	0.00
Haplo16	.AC..TAGT....CC.GA.ACT**G**GCGCGGT	0.01	0.01	0.01	0.00	0.03	0.00	0.05

**a** Haplo1 is the typical haplotype of Erhualian pigs that carries the derived *32E* allele; Haplo2 and Haplo3 represent European haplotypes for their predominant presence in European commercial and local breeds and wild boars; Haplo4 is an ancient haplotype that was evidenced in both Chinese and European wild boars.

Haplo9 and Haplo15 are two additional ancient haplotype mainly pertain to Chinese wild boars. The PPARD G32E alleles are indicated in bold.

### Diverse pieces of evidence support the casualty of *PPARD* G32E for the QTL

The elucidation of the genetic basis of multifactorial traits in domestic animals is still a big challenge, and few successful examples have been reported [23]–[27]. In this study, a battery of genetic and functional assays obtained diverse pieces of supporting evidence that the *PPARD* G32E substitution underlies the major QTL effect on ear size on SSC7. (1) The shared haloptypes of 9 F_1_ sires segregating for the QTL spanned a region of ∼1.2 Mb containing *PPARD*. (2) All Erhualian founder chromosomes shared a ∼750 kb segment spanning *PPARD* that were associated with the Q allele for increased ear size. (3) Erhualian pigs showed an obvious selective sweep signal in a 630-kb region encompassing *PPARD*; the signal was concordant with the breeding history of the breed. (4) The 630-kb haplotype showed similar QTL effect on increased ear size in Sutai pigs that were developed after 18-generation selection in the Erhualian × Duroc cross. (5) Of the 4 genes expressed in ear tissues within the critical region, *PPARD* stood out a prime candidate for its established essential roles in skin homeostasis, cartilage development and fat metabolism. (6) Only one missense mutation (G32E) was identified in *PPARD* using White Duroc and Erhualian founder animals. The mutation caused a nonconservative amino acid change at the conserved intrinsically disordered domain and was of functional significance. (7) The G32E SNP was concordant with QTL genotypes of F_0_ and F_1_ animals in the White Duroc × Erhualian cross. (8) The G32E SNP showed strikingly significant association with ear size across the experimental cross and diverse outbred populations. (9) The derived allele for increased ear size occurred at high frequencies only in Chinese floppy-eared breeds. Altogether, these data led us to conclude that G32E in the *PPARD* gene has an important contribution to ear size in pigs. The results establish, for the first time, a direct and novel role of *PPARD* in ear development and may be of relevance for the pathogenesis of external ear abnormalities in humans.

### Potential pleiotropic effects of *PPARD* G32E on diverse traits

The genomic region harboring *PPARD* G32E is of great interest in pig genetics, because significant QTL for diverse traits related to growth, carcass length, skeletal morphology and fat deposition have been consistently evidenced in the region using the current resource population and different crosses between Chinese Meishan and commercial breeds [28]–[33]. The overlapping QTL for multiple traits in the region led us to assume that there might be a single critical gene having pleiotrophic effects on these traits. We herein showed the causality of *PPARD* G32E for the QTL affecting ear size in the critical region. Given that *PPARD* serve as a crucial and multifaceted determinant of diverse biological functions including fat metabolism, cartilage development, chondrocyte proliferation and differentiation [8], [10]–[14], we thus speculate that *PPARD* is a strong candidate of the multiple significant QTL on SSC7 and that *PPARD* G32E might have pleiotropic effects on growth, carcass and fatness traits in pigs. Further investigations will be performed to validate the hypothesis in the future.

## Methods

### Ethics statement

All animal work was conducted according to the guidelines for the care and use of experimental animals established by the Ministry of Agriculture of China.

### Fine mapping by identical-by-descent analysis

Microsatellite markers in the mapped interval were mined from the pig genome assembly (Build 9.2) at http://www.ensembl.org/Sus_scrofa/Info/Index and were genotyped using standard procedures. Primers for amplification of microsatellite markers are given in Table S4. QTL genotypes of F_1_ boars in the White Duroc × Erhualian intercross were determined by marker-assisted segregation analysis as described previously [6]. Briefly, a Z-score was calculated for each F_1_ sire; the score is the log10 of the H_1_/H_0_ likelihood ratio where H_1_ assumes that the boar is heterozygous at the QTL (*Qq*), while H_0_ postulates that the boar is homozygous *QQ* or *qq*. Boars were considered to be *Qq* when *Z* >2, *QQ* or *qq* when *Z* <−2, and of undetermined genotype if −2<*Z*<2. The pedigree and management of the intercross population with phenotypic data of ear size have been described elsewhere [4]. Haplotypes of founder animals were reconstructed with the SimWalk2 program.

### Selective sweep detection

To detect the effects of a putative selection sweep on the genetic variation in Erhualian pigs compared with control animals, we analyzed the microsatellite and SNP genotypes of 211 Erhualian pigs and 335 control animals representing 10 different breeds (Hetao Large-Ear: 56; Laiwu: 32; Yushan Black: 31; Wuzhishan, 32; Dianan Small-Ear: 31; Tibetan: 34; White Duroc: 12; Duroc: 29; Large White: 39; Landrace: 39). SNP markers were genotyped using the ABI SNapshot protocol or PCR-RFLP assays. All primers are given in Table S4.

### RT-PCR of candidate genes in ear tissues

Total RNA was extracted from pig tissues using the Rneasy Fibrous Tissue Mini Kit (Qiagen). To analyze expression of candidate genes in ears, products from the first strand-complementary DNA synthesis (TaKaRa) were amplified with primers given in Table S5. The quantification of the *PPARD* transcripts was performed by the comparative C_t_ method (2^−ΔΔCt^) using the primers and TaqMan probes shown in Table S5. Real-time PCR was done with the Universal PCR Master Mix using an ABI7900 instrument (Applied Biosystem). All samples were analyzed in triplicate. The *β-actin* gene was used as the internal reference gene.

### Resequencing of *PPARD* cDNA and genotyping of *PPARD* G32E

The entire coding region of porcine *PPRAD* was re-sequenced using ear mRNA of two White Duroc and two Erhualian animals. Primer pairs listed in Table S6 were used to generate overlapping PCR amplicons. All PCR products were purified using the NucleoSpin Extract II kit (Macherey-Nagel) and sequenced using the same primers. The sequence traces were assembled and analyzed for polymorphisms using the SeqMan program (DNASTAR). The *PPARD* G32E mutation was genotyped using the ABI SNapshot protocol. A 385-bp DNA fragment was amplified with the F2/R2 primer pairs (F2: 5′-CGG CTG TTT TAC AGG AAG GA-3′; R2: 5′- CTG CAC TCA GAC CCA GAT GA-3′). SNapshot reactions were performed with Multiplex Ready Reaction Mix (Applied Biosystem) and an extension primer (5′-TTT TTT TTT TGC TGG AGG GAA GCG AGT GCT CTG GT -3′) using an ABI 3130XL DNA Analyzer (Applied Biosystem).

### Luciferase report assay

The coding region of pig *PPARD* was amplified with primers PPARD-Age-I-F (5′- GAG GAT CCC CGG GTA CCG GTC GCC ACC ATG GAG CAG CCG CCG GAG-3′) and PPARD-Age-I-R (5′- TCA TCC TTG TAG TCG CTA GCG TAC ATG TCC TTG TAG-3′). The amplified cDNA was gel-purified and digested with *Age*I and *Nhe*I (NEB). The restricted fragments were cloned to pGC-FU-EGFP-3FLAG lentiviral expression vector (Genechem). The sequence and orientation of the insert were verified by DNA sequencing. The expression of His-tagged PPARD in cultured cells was confirmed by Western blot analysis with anti-His antibody. The human 293T cells were infected with the lentiviral expression constructs of pig wild-type and mutant PPARD. The infected cells were seeded at a concentration achieving 80% confluence in 96-well plates 18 h before transfection. The cells were transiently transfected with TCF/LEF-Luc reporter vector (Cignal, SAB) along with a control *Relina* luciferase vector using Lipofectamine plus reagent. The cell lysates were obtained with 1× reporter lysis buffer (Promega) 48 h after transfection. The luciferase activity was assayed in a Berthold Auto Lumat LB953 luminometer (Nashua, NH) by using the luciferase assay system from Promega. The relative luciferase activity was normalized to the *Relina* luciferase activity in each sample.

### Real-time RT-PCR and western blot analysis in cultured cells

The pig ear-derived fibroblast cells were transfected with pGC-FU-EGFP-3FLAG lentiviral expression vector (Genechem). Five days post-transfection, 1×10^6^ cells were harvested for qPCR and Western blot analysis. Total RNA was extracted from harvested cells using Trizol (Invitrogen). Two µg of total RNA was synthesized into cDNA with M-MLV reverse transcriptase (Promega) and oligo d(T). Real time PCR was performed on the cDNA using the SYBR Premix Ex Taq (TaKaRa) and primers listed in Table S7 in a TP800 Real Time System (TaKaRa). The quantification of transcripts was performed by the comparative C_t_ (2^−ΔΔCt^) method. All values were reported as mean ± S.D. of triplicate assays of each cDNA sample. Rabbit anti-PPARD (Sigma), mouse anti-β-catenin (Abcam), rabbit anti-c-myc (Cellsignaling), mouse anti-Sox9 (Abcam) and mouse anti-GAPDH (Santa Cruz) antibodies were used in Western blots in a routine way. The specific immunoreactive bands were visualized using an ECL plus kit (GE Healthcare) and quantified with the Molecular Imaging Software (Kodak).

### Association analysis

The entire White Duroc × Erhualian resource population was genotyped for the *PPARD* G32E mutation. Association of the mutation with ear size and weight was evaluated using standard association, marker-assisted association and *F*-drop test as described previously [20]. Association analyses were also performed on 667 animals representing 7 different breeds. Photographs were taken for one ear of each animal after the ear was fixed and covered with a ruler as an internal reference of the size. Ear size was calculated using the Qwin software (Laica). Significance was evaluated by the *t*-test in the GLM procedure of SAS 9.0.

### Analysis of haplotype phylogenies and linkage disequilibrium

Genomic DNA pools of White Duroc (n = 2) and Erhualian (n = 2) animals were amplified with primers given in Table S6. All PCR products were purified with the Qiagen protocol and sequenced using the same PCR primers, revealing a subset of SNP markers in the genomic region of porcine *PPARD*. SNP markers were genotyped by iPLEX SEQUENOM MassARRAY platform. SNP genotype calls were filtered and checked manually, and aggressive calls were omitted from the dataset. Population genetics parameters including the mean number of pairwise differences across loci (π_N_), Tajimas *D*, Fu and Li's *D* were estimated with DnaSP v5 [34]. Haplotypes were reconstructed with PHASE v2 [35]. Haplotype phylogenetic tree based on *p*-distance were drawn using MEGA4 [36]. The Haploview v4.1 program [37] was used to calculate linkage disequilibrium measures (*r^2^* and *D'*) and to identify haplotype blocks.

## Supporting Information

Figure S1 Plots of *F*-ratios indicating the major QTL for ear size at 58 cM on pig chromosome 7. Markers and distance in cM are given on the x-axis, and *F*-ratios are indicated on the left y-axis. The threshold for 1% genome-wide significant level is indicated by the dashed horizontal line. The confidence interval of 2 cM is marked by the dashed vertical line. LEW: left ear weight; REW: right ear weight; LEA: left ear area; REA: right ear area.
(TIF)

Figure S2 QTL genotypes of F_1_ boars determined by marker-assisted segregation analysis. The number of offspring in each sire family is given above the error bars. The right ear size measured in the pedigree is marked by cm^2^ in left axis. A Z-score is given for each sire pedigree. *Q* alleles associated with increased ear size are marked by a diamond, *q* alleles by a circle.
(TIF)

Figure S3 Association of the Erhualian-originated haplotype in the critical 630-kb region with increased ear size in Sutai pigs. Rare haplotypes with frequencies of less than 0.01 were discarded for analysis. E denotes the Erhualian haplotype.
(TIF)

Figure S4 Real-time RT-PCR analysis of *PPARD* temporal expression in ear tissues of Erhualian and Duroc pigs. Tissue samples were collected from Erhualian and Duroc pigs at days 0, 45±3, 90±3, and 300±3 for RNA extraction. Six animals were sampled from each breed at each period. Real- time PCR was performed in triplicate. *PPARD* expression levels normalized with *β-actin* are given (mean ± s.e.). No significant difference was observed in *PPARD* expression levels between Erhualian and Duroc pigs at each stage.
(TIF)

Figure S5 Relationship between Tajima' *D* and frequency of the derived *32E* allele.
(TIF)

Figure S6 NJ phylogenetic tree constructed with the 16 frequent *PPARD* haplotypes. The detail information about each haplotype is given in Table 4. Haplotype 1 is the only one containing the derived *32E* allele for increased ear size and is the major haplotype of Erhualian pigs.
(TIF)

Figure S7 Linkage disequilibrium (*r^2^*) plot between pairs of loci for Chinese indigenous breeds (A), Erhualian pigs (B) and Western commercial breeds (C). Haplotype blocks are underlined, and the G32E locus is indicated by arrows.
(TIF)

Figure S8 Distribution of linkage disequilibrium measures (*r^2^* and *D'*) against the distance between the G32E mutation and the rest of loci.
(TIF)

Table S1 Descriptive statistics of the ear traits measured in the White Duroc × Erhualian cross.
(DOC)

Table S2 Effect of the *PPARD* G32E substitution on ear size in the White Duroc × Erhualian cross.
(DOC)

Table S3 Genetic variability in the *PPARD* gene in worldwide pig breeds.
(DOC)

Table S4 Primers for detection of SNP and microsatellite markers in the QTL region.
(DOC)

Table S5 Primers for analyzing expression of annotated genes in the refined interval using RT-PCR and real-time PCR.
(DOC)

Table S6 Primers for identification of polymorphisms in the coding and genomic regions of porcine *PPARD*.
(DOC)

Table S7 Primers for real time RT-PCR analysis in cultured cells.
(DOC)

## References

[1] Alasti F, Van Camp G (2009). Genetics of microtia and associated syndromes.. J Med Genet.

[2] Andersson L, Georges M (2004). Domestic-animal genomics: deciphering the genetics of complex traits.. Nat Rev Genet.

[3] Zhang ZG, Li BD, Chen XH (1986). *Pig Breeds in China*..

[4] Ma J, Qi W, Ren D, Duan Y, Qiao R (2009). A genome scan for quantitative trait loci affecting three ear traits in a White Duroc × Chinese Erhualian resource population.. Anim Genet.

[5] Wei WH, de Koning DJ, Penman JC, Archibald AL, Haley CS (2007). QTL modulating ear size and erectness in pigs.. Anim Genet.

[6] Nezer C, Collette C, Moreau L, Brouwers B, Kim JJ (2003). Haplotype sharing refines the location of an imprinted quantitative trait locus with major effect on muscle mass to a 250-kb chromosome segment containing the porcine *IGF2* gene.. Genetics.

[7] Shen ZL (2002). A newly developed pig breed: Sutai pigs [in Chinese].. Swine Prod.

[8] Barish GD, Narkar VA, Evans RM (2006). PPARδ: a dagger in the heart of the metabolic syndrome.. J Clin Invest.

[9] Di-Poï N, Michalik L, Desvergne B, Wahli W (2004). Functions of peroxisome proliferator-activated receptors (PPAR) in skin homeostasis.. Lipids.

[10] Wang YX, Lee CH, Tiep S, Yu RT, Ham J (2003). Peroxisome-proliferator-activated receptor delta activates fat metabolism to prevent obesity.. Cell.

[11] Evans RM, Barish GD, Wang YX (2004). PPARs and the complex journey to obesity.. Nat Med.

[12] Wang YX, Zhang CL, Yu RT, Cho HK, Nelson MC (2004). Regulation of muscle fiber type and running endurance by PPARdelta.. PLoS Biol.

[13] Han C, Lim K, Xu L, Li G, Wu T (2008). Regulation of Wnt/β-catenin pathway by cPLA_2_α and PPARδ.. J Cell Biochem.

[14] Macsai C, Foster BK, Xian C (2007). Roles of Wnt signaling in bone growth, remodeling, skeletal disorders and fracture repair.. J Cell Physiol.

[15] Haynes C, Oldfield CJ, Ji F, Klitgord N, Cusick ME (2006). Intrinsic disorder is a common feature of hub proteins from four eukaryotic interactomes.. PLoS Comput Biol.

[16] Li YJ, Wei ZM, Meng YX, Ji XR (2005). Beta catenin up-regulates the expression of cyclinD1, c-myc and MMP-7 in human pancreatic cancer: relationships with carcinogenesis and metastasis.. World J Gastroenterol.

[17] Topol L, Chen W, Song H, Day TF, Yang Y (2009). Sox9 inhibits Wnt signaling by promoting beta-catenin phosphorylation in the nucleus.. J Biol Chem.

[18] Ross SE, Hemati N, Longo KA, Bennett CN, Lucas PC (2000). Inhibition of adipogenesis by Wnt signaling.. Science.

[19] Bennett CN, Ross SE, Longo KA, Bajnok L, Hemati N (2002). Regulation of Wnt signaling during adipogenesis.. J Biol Chem.

[20] Zhao H, Rothschild MF, Fernando RL, Dekkers JCM (2003). Test of candidate genes in breed cross populations for QTL mapping in livestock.. Mamm Genome.

[21] Amaral AJ, Megens HJ, Crooijmans RP, Heuven HC, Groenen MA (2008). Linkage disequilibrium decay and haplotype block structure in the pig.. Genetics.

[22] Kijas JM, Wales R, Törnsten A, Chardon P, Moller M (1998). Melanocortin receptor 1 (*MC1R*) mutations and coat color in pigs.. Genetics.

[23] Van Laere AS, Nguyen M, Braunschweig M, Nezer C, Collette C (2003). A regulatory mutation in *IGF2* causes a major QTL effect on muscle growth in the pig.. Nature.

[24] Grisart B, Farnir F, Karim L, Cambisano N, Kim JJ (2004). Genetic and functional confirmation of the causality of the *DGAT1* K232A quantitative trait nucleotide in affecting milk yield and composition.. Proc Natl Acad Sci USA.

[25] Clop A, Marcq F, Takeda H, Pirottin D, Tordoir X (2006). A mutation creating a potential illegitimate microRNA target site in the myostatin gene affects muscularity in sheep.. Nat Genet.

[26] Sutter NB, Bustamante CD, Chase K, Gray MM, Zhao K (2007). A single IGF1 allele is a major determinant of small size in dogs.. Science.

[27] Rubin CJ, Zody MC, Eriksson J, Meadows JR, Sherwood E (2010). Whole-genome resequencing reveals loci under selection during chicken domestication.. Nature.

[28] Rohrer GA, Keele JW (1998). Identification of quantitative trait loci affecting carcass composition in swine: I. Fat deposition traits.. J Anim Sci.

[29] Rohrer GA (2000). Identification of quantitative trait loci affecting birth characters and accumulation of backfat and weight in a Meishan-White Composite resource population.. J Anim Sci.

[30] de Koning DJ, Janss LL, Rattink AP, van Oers PA, de Vries BJ (1999). Detection of quantitative trait loci for backfat thickness and intramuscular fat content in pigs (*Sus scrofa*).. Genetics.

[31] Bidanel JP, Milan D, Iannuccelli N, Amigues Y, Boscher MY (2001). Detection of quantitative trait loci for growth and fatness in pigs.. Genet Sel Evol.

[32] Sato S, Oyamada Y, Atsuji K, Nade T, Sato S (2003). Quantitative trait loci analysis for growth and carcass traits in a Meishan × Duroc F2 resource population.. J Anim Sci.

[33] Mao H, Guo Y, Yang G, Yang B, Ren J (2008). A genome-wide scan for quantitative trait loci affecting limb bone lengths and areal bone mineral density of the distal femur in a White Duroc × Erhualian F_2_ population.. BMC Genet.

[34] Librado P, Rozas J (2009). DnaSP v5: A software for comprehensive analysis of DNA polymorphism data.. Bioinformatics.

[35] Stephens M, Donnelly P (2003). A comparison of Bayesian methods for haplotype reconstruction from population genotype data.. Am J Hum Genet.

[36] Tamura K, Dudley J, Nei M, Kumar S (2007). MEGA4: Molecular Evolutionary Genetics Analysis (MEGA) software version 4.0.. Mol Biol Evol.

[37] Barrett JC, Fry B, Maller J, Daly MJ (2005). Haploview: analysis and visualization of LD and haplotype maps.. Bioinformatics.

