# Supplementary figures and images for: A Missense Mutation in PPARD Causes a Major QTL Effect on Ear Size in Pigs
Source: PLoS Genet. 2011 May 5;7(5):e1002043. doi: 10.1371/journal.pgen.1002043 (PMC3088719; doi:10.1371/journal.pgen.1002043)

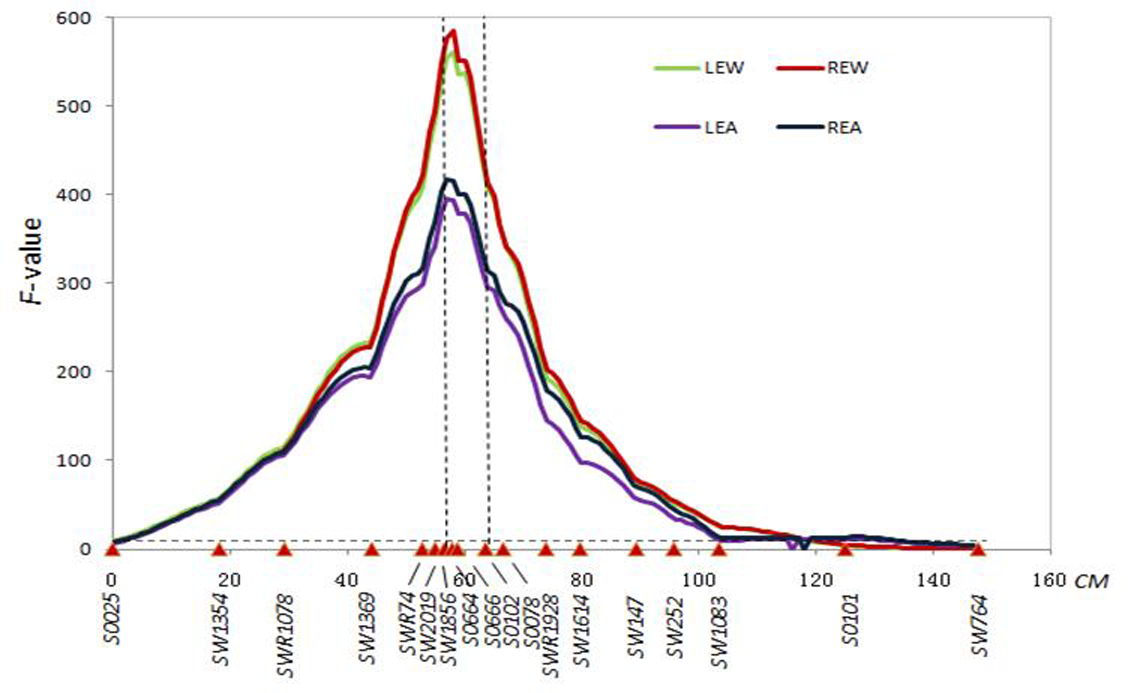

Supplement: Figure S1 — Plots of F-ratios indicating the major QTL for ear size at 58 cM on pig chromosome 7. Markers and distance in cM are given on the x-axis, and F-ratios are indicated on the left y-axis. The threshold for 1% genome-wide significant level is indicated by the dashed horizontal line. The confidence interval of 2 cM is marked by the dashed vertical line. LEW: left ear weight; REW: right ear weight; LEA: left ear area; REA: right ear area. (TIF) [file pgen.1002043.s001.tif]

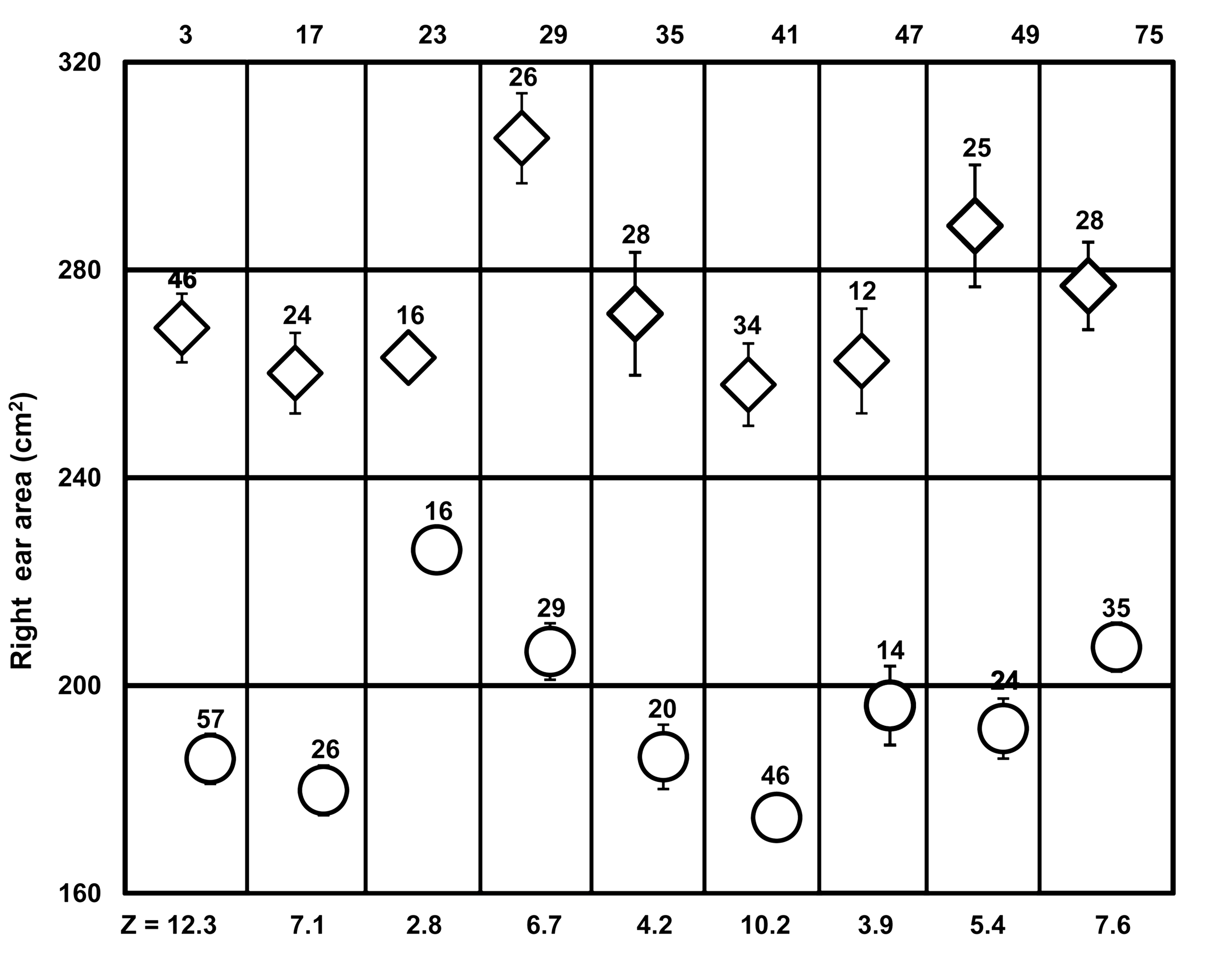

Supplement: Figure S2 — QTL genotypes of F1 boars determined by marker-assisted segregation analysis. The number of offspring in each sire family is given above the error bars. The right ear size measured in the pedigree is marked by cm2 in left axis. A Z-score is given for each sire pedigree. Q alleles associated with increased ear size are marked by a diamond, q alleles by a circle. (TIF) [file pgen.1002043.s002.tif]

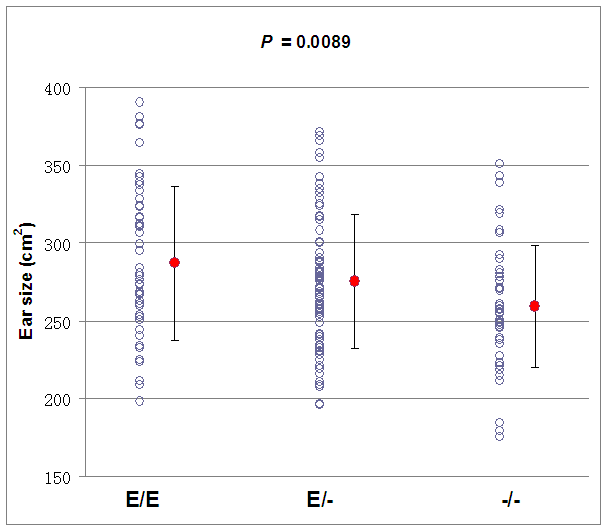

Supplement: Figure S3 — Association of the Erhualian-originated haplotype in the critical 630-kb region with increased ear size in Sutai pigs. Rare haplotypes with frequencies of less than 0.01 were discarded for analysis. E denotes the Erhualian haplotype. (TIF) [file pgen.1002043.s003.tif]

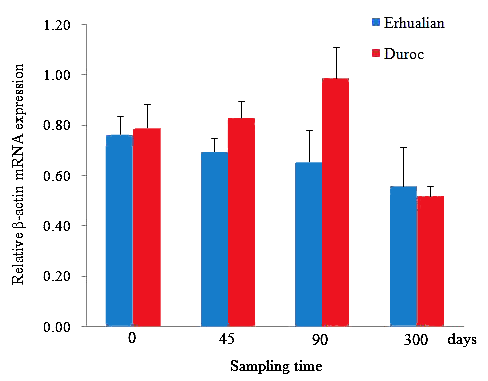

Supplement: Figure S4 — Real-time RT-PCR analysis of PPARD temporal expression in ear tissues of Erhualian and Duroc pigs. Tissue samples were collected from Erhualian and Duroc pigs at days 0, 45±3, 90±3, and 300±3 for RNA extraction. Six animals were sampled from each breed at each period. Real- time PCR was performed in triplicate. PPARD expression levels normalized with β-actin are given (mean ± s.e.). No significant difference was observed in PPARD expression levels between Erhualian and Duroc pigs at each stage. (TIF) [file pgen.1002043.s004.tif]

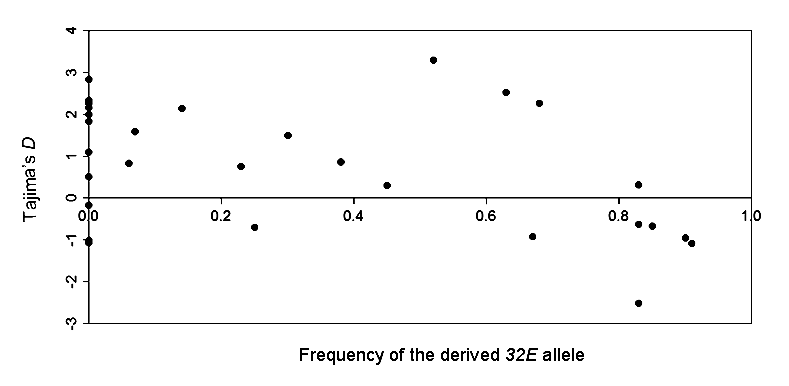

Supplement: Figure S5 — Relationship between Tajima' D and frequency of the derived 32E allele. (TIF) [file pgen.1002043.s005.tif]

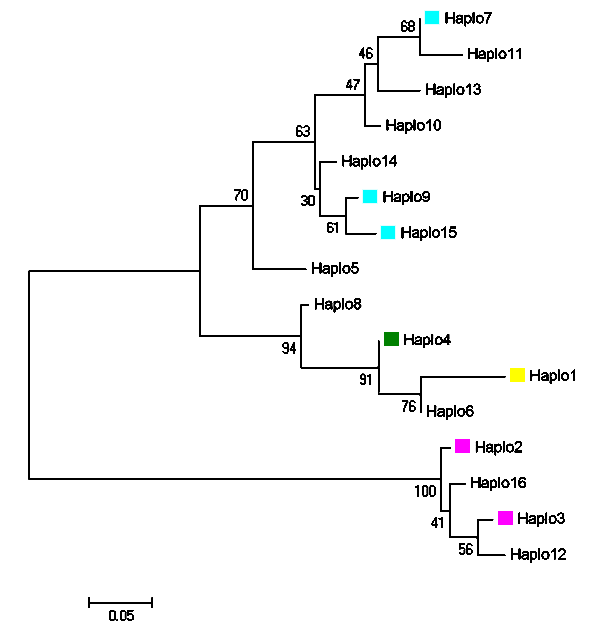

Supplement: Figure S6 — NJ phylogenetic tree constructed with the 16 frequent PPARD haplotypes. The detail information about each haplotype is given in Table 4. Haplotype 1 is the only one containing the derived 32E allele for increased ear size and is the major haplotype of Erhualian pigs. (TIF) [file pgen.1002043.s006.tif]

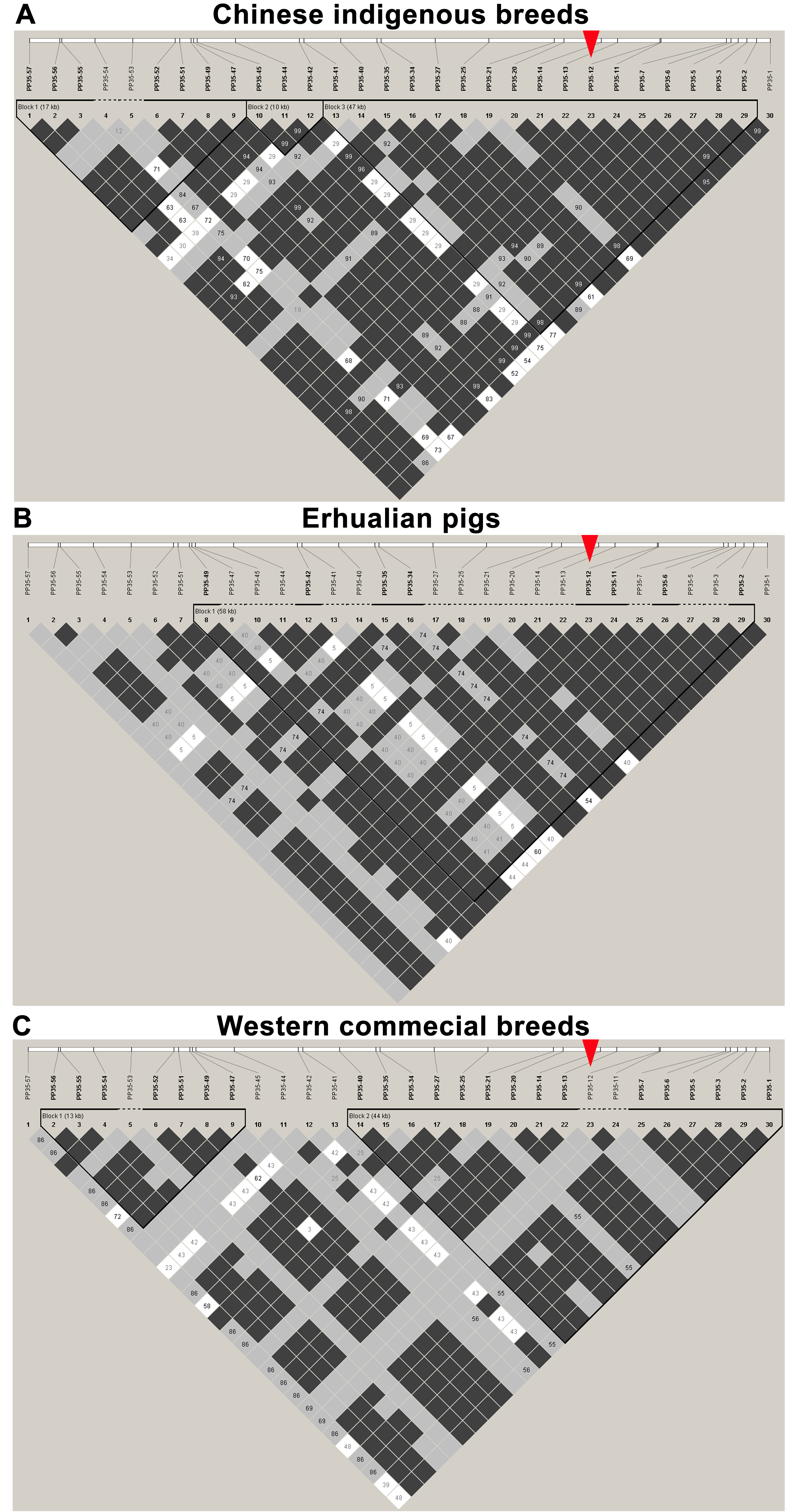

Supplement: Figure S7 — Linkage disequilibrium (r2) plot between pairs of loci for Chinese indigenous breeds (A), Erhualian pigs (B) and Western commercial breeds (C). Haplotype blocks are underlined, and the G32E locus is indicated by arrows. (TIF) [file pgen.1002043.s007.tif]

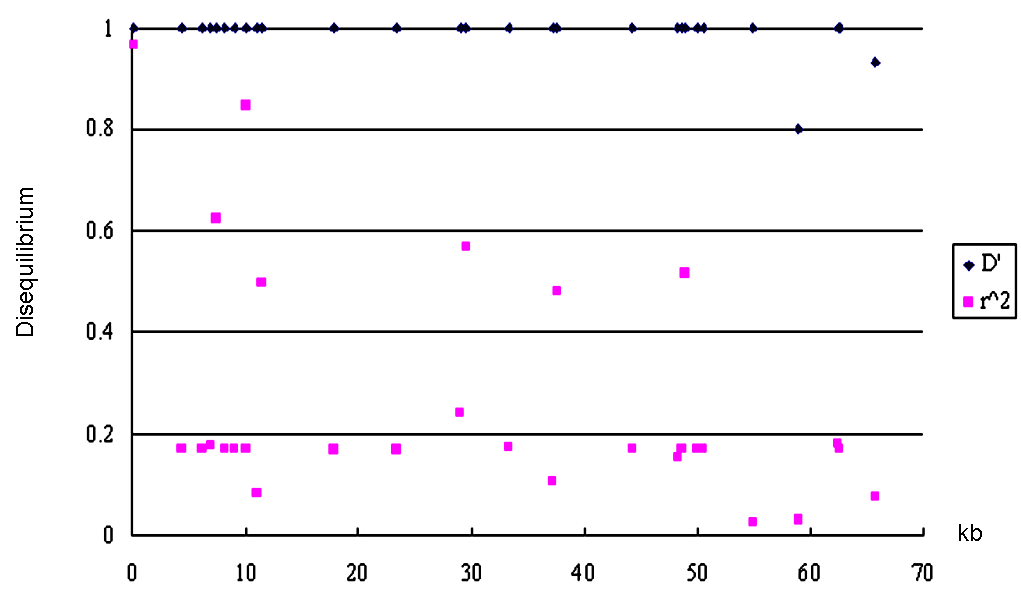

Supplement: Figure S8 — Distribution of linkage disequilibrium measures (r2 and D') against the distance between the G32E mutation and the rest of loci. (TIF) [file pgen.1002043.s008.tif]
