# Supplementary material for: A Missense Mutation in PPARD Causes a Major QTL Effect on Ear Size in Pigs
Source: PLoS Genet. 2011 May 5;7(5):e1002043. doi: 10.1371/journal.pgen.1002043 (PMC3088719; doi:10.1371/journal.pgen.1002043)
Supplement: Table S1 — Descriptive statistics of the ear traits measured in the White Duroc × Erhualian cross. (DOC) [file pgen.1002043.s009.doc]

**Supplementary Table 1** Descriptive statistics of the ear traits measured in the White Duroc × Erhualian cross ^a^

| Trait | Ear side | No. | Mean | SD | Minimum | Maximum |
| --- | --- | --- | --- | --- | --- | --- |
| weight (g) | Left | 1027 | 249.82 | 84.98 | 72.50 | 692.50 |
|  | Right | 1027 | 257.76 | 85.44 | 100.00 | 605.00 |
| Area (cm^2^) | Left | 1013 | 239.36 | 52.15 | 98.55 | 432.73 |
|  | Right | 1012 | 236.33 | 50.56 | 88.91 | 429.23 |
| Erectness |  | 1622 | 1.89 | 0.83 | 1.00 | 3.00 |

^a^ This table is quoted from Ma et al. (2009).
