# Supplementary material for: A Missense Mutation in PPARD Causes a Major QTL Effect on Ear Size in Pigs
Source: PLoS Genet. 2011 May 5;7(5):e1002043. doi: 10.1371/journal.pgen.1002043 (PMC3088719; doi:10.1371/journal.pgen.1002043)
Supplement: Table S2 — Effect of the PPARD G32E substitution on ear size in the White Duroc × Erhualian cross. (DOC) [file pgen.1002043.s010.doc]

**Supplementary Table 2** Effect of the *PPARD* G32E substitution on ear size in the White Duroc × Erhualian F_2_ cross ^a^

| Trait | Number | Model | | | Explained variance of the QTL effect (%) |
| --- | --- | --- | --- | --- | --- |
|  |  | Standard association  (*P* value) | Marker-assisted association  (*P* value) | *F*-drop test |  |
| Ear weight | 1027 | <0.0001 | 9.25 × 10^-6^ | 0.02 | 98 |
| Ear size | 1013 | <0.0001 | 3.16 × 10^-3^ | 0.03 | 98 |

^a^ Computed as described in Zhao et al. (2003). Briefly, we used 4 models in the association tests: y = μ + SNP + fixed effects + covariate + e (model 1, SNP model); y = μ + fixed effects + covariate + e (model 2, reduced model); y = μ + QTL + SNP + fixed effects + covariate + e (model 3, QTL +SNP model); y = μ + QTL + fixed effects + covariate + e (model 4, QTL model); where y is the trait value, μ is the overall mean, e represents the residual effect. Sex and batch were included as fixed effects and carcass weight as a covariate in the models. Significance was tested based on *F* ratios of residual sums of squares for models 1 and 2 (Standard association), ratios of residual sums of squares for models 3 and 4 (Marker-assisted association), or by comparing the *F* ratio of residual sums of squares for models 1 and 3 to the *F* ratio for models 2 and 4 (*F*-drop test).
