# Supplementary material for: A Missense Mutation in PPARD Causes a Major QTL Effect on Ear Size in Pigs
Source: PLoS Genet. 2011 May 5;7(5):e1002043. doi: 10.1371/journal.pgen.1002043 (PMC3088719; doi:10.1371/journal.pgen.1002043)
Supplement: Table S3 — Genetic variability in the PPARD gene in worldwide pig breeds. (DOC) [file pgen.1002043.s011.doc]

**Supplementary Table 3** Genetic variability in the *PPARD* gene in worldwide pig breeds ^a^

| Breeds | Locality | N | S | *P*_A_ ^b^ | π_N_ | *D*_T_ | *D*_FL_ |
| --- | --- | --- | --- | --- | --- | --- | --- |
| Chinese local breeds |  | 1262 | 30 | 0.55 | 0.22 | 1.78 | 1.96 |
| Bama Xiang | Guangxi | 58 | 29 | 0.30 | 0.30 | 1.49 | 1.85 |
| Erhualian | Changshu | 142 | 30 | 0.91 | 0.14 | -0.68 | 1.62 |
| Erhualian | Wujin | 134 | 29 | 0.85 | 0.12 | -0.95 | 1.96 |
| Erhualian | Xishan | 134 | 0 | 0.90 | 0 | - | - |
| Erhualian | ALL | 410 | 30 | 1.00 | 0.09 | -1.09 | 2.02 |
| Hangzhu | Jiangxi | 58 | 14 | 0.63 | 0.19 | 2.52 | 0.54 |
| Hetao | Neimeng | 60 | 27 | 0.83 | 0.16 | -0.63 | 1.82 |
| Jiangquhai | Jiangsu | 60 | 15 | 0.07 | 0.16 | 1.58 | 1.58 |
| Jinhua | Zhejiang | 58 | 10 | 0.00 | 0.05 | -1.01 | -2.41 |
| Laiwu | Shandong | 58 | 26 | 0.00 | 0.25 | 1.09 | 1.81 |
| Lantang | Guangdong | 60 | 3 | 0.83 | 0.02 | 0.32 | 0.87 |
| Meishan | Shanghai | 28 | 23 | 0.52 | 0.38 | 3.29 | 1.66 |
| Minzhu | Heilongjiang | 60 | 22 | 0.67 | 0.11 | -0.92 | 1.38 |
| Ningxiang | Hunan | 44 | 15 | 0.14 | 0.19 | 2.14 | 1.56 |
| Rongchang | Chongqing | 58 | 16 | 0.38 | 0.15 | 0.86 | 0.69 |
| Shaziling | Hunan | 16 | 28 | 0.06 | 0.34 | 0.83 | 1.08 |
| Taihu | Jiangsu | 22 | 23 | 0.83 | 0.07 | -2.52 | -4.02 |
| Tongcheng | Hubei | 58 | 29 | 0.45 | 0.23 | 0.3 | -2.5 |
| Wuzhishan | Hainan | 54 | 29 | 0.23 | 0.26 | 0.75 | 1.83 |
| Yushan Heizhu | Jiangxi | 46 | 23 | 0.68 | 0.29 | 2.26 | 1.73 |
| Zangzhu | Tibet | 54 | 27 | 0.00 | 0.23 | 0.51 | 1.81 |
| Wild boar |  | 42 | 12 | 0.00 | 0.15 | 1.87 | 1.48 |
| Commercial breeds |  | 268 | 28 | 0.00 | 0.32 | 3.01 | 1.10 |
| Duroc |  | 50 | 22 | 0.00 | 0.26 | 1.84 | 0.35 |
| Landrace |  | 64 | 26 | 0.00 | 0.31 | 2.16 | 1.49 |
| Large White |  | 132 | 28 | 0.00 | 0.31 | 2.33 | 1.19 |
| White Duroc |  | 22 | 22 | 0.00 | 0.35 | 2.84 | 1.61 |
| Western local breeds |  | 54 | 29 | 0.02 | 0.33 | 1.94 | 1.27 |
| Bershire | USA | 8 | 16 | 0.00 | 0.29 | 2 | 1.56 |
| British Lop | UK | 2 | - | 0.00 | - | - | - |
| Chester White | USA | 2 | - | 0.00 | - | - | - |
| Hampshire | USA | 2 | - | 0.00 | - | - | - |
| Iberian | Spain | 20 | 20 | 0.00 | 0.13 | -1.08 | 1.58 |
| Large Black | UK | 4 | 2 | 0.25 | 0.03 | -0.71 | -0.71 |
| Mid White | UK | 4 | 23 | 0.00 | 0.41 | -0.17 | -0.17 |
| Old Spot | UK | 4 | 16 | 0.00 | 0.36 | 2.26 | 2.26 |
| Saddle Black | UK | 2 | - | 0.00 | - | - | - |
| Tamwork | UK | 2 | - | 0.00 | - | - | - |
| Yorkshire | UK | 4 | 0 | 0.00 | 0 | - | - |
| Wild boar | EU | 20 | 20 | 0.00 | 0.08 | -2.14 | -3.22 |

^a^ N, number of haplotypes; S, number of segregating sites; P_A_, the frequency of derived allele; π_N_, mean number of pair wise differences across SNPs; *D*_T_, Tajima’s *D*; *D*_FL_, Fu and Li’s *D* index; *D* indexes are reported when N≧4.

^b^ P^A^ values in this table is slightly different from those in Table 3 due to different sample size per breed.
