# Supplementary material for: A Missense Mutation in PPARD Causes a Major QTL Effect on Ear Size in Pigs
Source: PLoS Genet. 2011 May 5;7(5):e1002043. doi: 10.1371/journal.pgen.1002043 (PMC3088719; doi:10.1371/journal.pgen.1002043)
Supplement: Table S4 — Primers for detection of SNP and microsatellite markers in the QTL region. (DOC) [file pgen.1002043.s012.doc]

**Supplementary Table 4** Primers for detection of SNP and microsatellite markers in the QTL region.

| Locus | Amplification primer (5’-3’) ^a^ | SNapshot primer (5’-3’) or restriction enzyme^b^ | Amplicon (bp) | Tm (^o^C) |
| --- | --- | --- | --- | --- |
| *ANKS1A* g.204A>G | F: GCCAGCTTTTCTCTGGCAT | GAGGGCTGGTGACACGCAGGA | 236 | 65 |
|  | R: GCTTCATAACCGCAGGACTCA |  |  |  |
| *C6orf106* g.410A>G | F: CTTCATTTCGACCCCACAAT | TAGCAGTGGAGAAGTCATATAATAG | 361 | 61 |
|  | R: CCCAAGTGACCACTGAGGTT |  |  |  |
| *CLPS* g.347T>G | F: GGGTTCAGAGGACTTCTCC | GGAGCCAGTGGAGCCACAGG | 581 | 64 |
|  | R: CCTGTTCTAGGCCACATATG |  |  |  |
| *CLPS* g.2151C>G | F: GGGTTCAGAGGACTTCTCC | TATTAACTTGATCTCTCTGGGGAGCCT | 581 | 64 |
|  | R: CCTGTTCTAGGCCACATATG |  |  |  |
| *COL21A1* g.34470T>C | F: AAGCATATGTCCTGGTGAC | *Cpo* I | 743 | 54 |
|  | R: GACAGGTTACACCCCAATAC |  |  |  |
| *COL21A1* g.54076 T>A | F: GGCTAGTTGCAAGCAAATATTG | *Ssp* I | 166 | 64 |
|  | R: CAGGCCTACCTCACCATTCT |  |  |  |
| *COL21A1* g.145976_145077insG | F: CAACCAAGTTGTAAGGGCTTTG | *BamH* I | 810 | 61 |
|  | R: GGTCGCTGGCTTTCAGATAC |  |  |  |
| *FANCE* g.585G>A | F: CCCACAGCCTGAGGTAGAAG | GGGGAGGGAGCCCAGGGT | 186 | 63 |
|  | R: CTCAGCACCTCATCCTGACC |  |  |  |
| *FKBP5* g.797A>C | F: CATTTTGTGCCAGAGACACG | GGCTTCTTGAATTGTTTCCC | 239 | 65 |
|  | R: TCCCTCGAATGCAACTCTCT |  |  |  |
| *FKBP5* g.1821T>A | F: AGACGAGGAAGAGCAAGCAG | GCTGTGACAAGGTAAAGTG | 201 | 65 |
|  | R: CGGCTTGTCAGAAAAGGAGT |  |  |  |
| *HMGA1* g.2255T>C | F: TCTGCCTCCACTCTCTAAAAGG | *Msl* I | 387 | 54 |
|  | R: AGTAACCCAACACCCCTTCTG |  |  |  |
| *HMGA1* g.3135C>T | F: CGAAGTGCCAACACCTAAAAG | *Sph* I | 772 | 60 |
|  | R: TGGAGCTGTGGTGGTTTTC |  |  |  |
| *HMGA1* g.4262G>A | F: CCATTGGGTGAGCACTGTC | *Hha* I | 498 | 65 |
|  | R: CTAGAGGAAGGCCCTGGTG |  |  |  |
| *NUDT3* g.423T>G | F: GATGGGAAGTCCAGGATCAA | CAAAGAGAGACAGAAGAGTGGACG | 231 | 59 |
|  | R: GCTCCCCCTCCAAATCCGTA |  |  |  |
| *PACSIN1* g.434T>C | F: CATGGCACAGTTGACCTAG | ACGTGGGGGGCTCTGGAGGG | 164 | 65 |
|  | R: ACAGCTTCTGGGAGGTGAGG |  |  |  |
| *PACSIN1* g.1689G>A | F: AGCCACCTGCTATTCTCAGC | AGAAGTATGAGAAGGTGCTGGACGAC | 303 | 60 |
|  | R: AAGCAGGATGTGCAGAAGGT |  |  |  |
| *PACSIN1* g.2566C>T | F: TCTTTGGTGGGATCTGAAGGT | GAGGCCAGGGTGGTGGGACT | 226 | 65 |
|  | R: CTGCCCTCACCATAGAGTTC |  |  |  |
| *PPARD* g.61079C>T | F: CCCAAACCAAGTGCCAAGTG | *Bsa* I | 498 | 60 |
|  | R: TCTGACCCACAATGATGCTG |  |  |  |
| *PPARD* g.61399G>A | F: CGGCTGTTTTACAGGAAGGA | GCTGGAGGGAAGCGAGTGCTCTGGT | 385 | 63 |
|  | R: CTGCACTCAGACCCAGATGA |  |  |  |
| *PPARD* g.61489C>G | F: GCCTGAGTGCTGGTGGTTAT | AGGCGGGAGCCTGGCCCGG | 712 | 60 |
|  | R: TCTGACCCACAATGATGCTG |  |  |  |
| *PPARD* g.70478G>A | F: GAGGAATTGCTCCATCCTGA | CAGGTCGGCCACTGGCCTT | 414 | 60 |
|  | R: GCAAATCAGGAAAAGCTTGG |  |  |  |
| *RPL10A* g.457C>T | F: TTTTGGGAAGGTTCAGCAAC | GCTCACAGCAACACCAGA | 199 | 61 |
|  | R: AACCATGAGGTTGCAGGTTC |  |  |  |
| *RPS10* g.29A>G | F: TGGTTAGTGTGGTGAAGA | GGTTAGTGTGGTGAAGAC | 190 | 54 |
|  | R: GGGATAAGACTGGATTGG |  |  |  |
| *SCUBE3* g.4358A>G | F: TCTGTTCCTCTTGGTCCGACTC | *Msp* I | 601 | 64 |
|  | R: GAGGATGAGACCTGGGGTACTG |  |  |  |
| *SCUBE3* g.14001C>T | F: CCGGTTTTTGCCAGGTACATAG | *Ase* I | 777 | 64 |
|  | R: TGCTGGAAGGCTGGGTAGTAAG |  |  |  |
| *SPDEF* g.7592C>T | F: CAGGGCTTCTGTGTAGTATGTGC | *Ava* II | 609 | 67 |
|  | R: CATCCCACTCTGACTCTGAACTC |  |  |  |
| *SRPK1* g.78A>T | F: ATCAGAAATGTGGCTTGGTTCT | *HpyCH4* IV | 452 | 56 |
|  | R: CCTCACCAATACAAACTTGTCG |  |  |  |
| *SRPK1* g.954G>A | F: GATTGCTGATCTTGGAAATGC | *Alu* I | 220 | 59 |
|  | R: TCCCATTATGGTTTATCACAGG |  |  |  |
| *SRPK1* g.1687A>G | F: CTTTCAAGGGTTTCTAGGGTTTG | *Mse* I | 158 | 64 |
|  | R: AGTCCGTTAAGTCTTTGGCAGA |  |  |  |
| *TAF11* g.86A>G | F: CCCAGGCTAGGGGTCTAATC | AGCCGCTGACCTACCCCACA | 347 | 61 |
|  | R: ATTGAGGAGTGGCAGCAAAT |  |  |  |
| *TCP11* g.828G>A | F: TTCTCCTTCTTGGCGATGTT | CCTCTGAGCAGCGACAACACAGC | 244 | 65 |
|  | R: GAGGGTGACCATGAGGACAG |  |  |  |
| *TULP1* g.409T>C | F: ATGTGGCTTGGATCTGGTGT | GCTTGGATCTGGTGTGGCTAC | 269 | 63 |
|  | R: TCCCCTCTGTCCTCCTCTA |  |  |  |
| *UHRF1BP1* g.476G>A | F: GCACAAAAGAGGGAGACCAC | ACTTTTATGTCACTGTGTACCCATGC | 257 | 64 |
|  | R: GGTTCAGTGAGGTGGCCTTA |  |  |  |
| *UHRF1BP1* g.1589T>C | F: CACAGCTGCATTTCTGCATT | CACTTCTATCAAACGGCCAGGA | 275 | 64 |
|  | R: TGACGTGTATTGGTGGCAGT |  |  |  |
| *ZNF76* g.324C>T | F: CCCTGTTTACTGGGTCTTATGC | *Hph* I | 547 | 65 |
|  | R: GTATGTGCTCTCTCATGCACCTG |  |  |  |
| *ZNF76* g.1218A>C | F: CTTCACCGAGTACTCAAGCCTAT | *BstU* I | 354 | 64 |
|  | R: TCTTTCACCTCTGACAGGTAAGC |  |  |  |
| *ZNF76* g.1917T>C | F: GTGCCACACAGGCATCCTT | *Nla* III | 432 | 54 |
|  | R: TCCAGAGAGGAGGTTATTGCTG |  |  |  |
| *GU565983* g.210T>A | F: CAGGCACCAAGGGCACTCAT | GCCAGGGTGTCCAGAGAGCAG | 390 | 65 |
|  | R: GGACCAGCAGCATCCACTCA |  |  |  |
| *GU565984* g.194A>G | F: TACGGCTGCACCTGAGACA | CTGGTGTTGCTGTGGCAGTGG | 744 | 65 |
|  | R: CACCCTGATAACCAGTGTTCTTG |  |  |  |
| *151L19-10S* g.241G>A | F: AGGTGAGAGAGATCCTTGCC | AGGCAACCTGAGGGAGGAG | 368 | 63 |
|  | R: ACAGGGATGCCTCTAAACGC |  |  |  |
| *191S* g.344A>G | F: TTGGGCTCCTGCTTAGTCT | GGGAGTGAGGAGCCGAGG | 1041 | 60 |
|  | R: GAACATCTTGTGCAACCGTAC |  |  |  |
| *SC701* | F: M13-TTGCTGTACGGCAGAACTTG |  | 154 - 160 | 65 |
|  | R: GTGTGGGAAGAAAAGCGAAGG |  |  |  |
| *SC702* | F: M13-GCTTCCTCTGACACACAGC |  | 199 - 212 | 65 |
|  | R: AAGAAGCAGCAAAATTTGAAG |  |  |  |
| *SC703* | F: M13-ACAGCAGACCAGTCATACATA |  | 224 - 232 | 60 |
|  | R: TGGAGTGGATAAGTAGTGAG |  |  |  |
| *SC704* | F: M13-TGGCTTTTCTGTGTGATTGC |  | 167 - 197 | 65 |
|  | R: AGGCGTCTCACTGTTTTGCT |  |  |  |
| *SC705* | F: M13-TGTGGTCCTTCCCTGGATAC |  | 173 - 186 | 65 |
|  | R: GCTTCCAGATCCTTCGTGAG |  |  |  |
| *SC706* | F: M13-CCCTTCTATGGCATATCTT |  | 254 - 289 | 65 |
|  | R: CAGGAAGCAAGCAGGTAAA |  |  |  |
| *SC707* | F: CCTCCTCCATTCCACAAAGA |  | 195 - 265 | 68 |
|  | R: GATCCATGCGTATTAGGGTCA |  |  |  |
| *SC708* | F: M13-GCGCTAATGTCTGTCCCAGT |  | 195 - 265 | 65 |
|  | R: CCCTTTGCCTGCTCTATCTG |  |  |  |
| *SC709* | F: M13-CAGAACTGATGGGGTGTGTG |  | 202 - 204 | 57 |
|  | R: AAGCTCTGCTCCAGACGAAC |  |  |  |
| *SC710* | F: M13-GATTATTCCTGGCTTGTC |  | 189 - 191 | 66 |
|  | R: GCCAACACTCAGCAGTAAGT |  |  |  |
| *SW1856* | F: TCATTCCAAACACACAGAGTCC |  | 170 -199 | 58 |
|  | R: TTGTATGGTATCCTGTGATGCC |  |  |  |
| *S0664* | F: AATCTTCCCTGTGGTTCATGG |  | 114 - 120 | 54 |
|  | R: CGGTGTTTGGGAGTAAATTCG |  |  |  |
| *S0665* | F: GGGCATCTGCAAATCTGATAA |  | 159 - 179 | 64 |
|  | R: AGCTTGAAGGCAGCTAGACG |  |  |  |
| *S0666* | F: GTGGATGTGGCCTTATGGTGAC |  | 195 - 205 | 65 |
|  | R: TTGCTGTGGCTCTGGCGTAG |  |  |  |
| *S0667* | F: GATCAGGGCCTGGTGTGT |  | 117 - 125 | 65 |
|  | R: TGGGAGAAAGGTTGGAGTTG |  |  |  |
| *S0671* | F: GCAGGCAGGAAGACATGAAT |  | 141 - 151 | 66 |
|  | R: CCTAACCTGGGAACCTCCAT |  |  |  |

^a^ M13 is the universal adapter of forward primer of each microsatellite marker and its sequence is GTTTTCCCAGTCACGACGTTG.

^b^ Restriction enzymes for genotyping SNP markers by PCR-RFLP assay are given.
