# Supplementary material for: A Missense Mutation in PPARD Causes a Major QTL Effect on Ear Size in Pigs
Source: PLoS Genet. 2011 May 5;7(5):e1002043. doi: 10.1371/journal.pgen.1002043 (PMC3088719; doi:10.1371/journal.pgen.1002043)
Supplement: Table S5 — Primers for analyzing expression of annotated genes in the refined interval using RT-PCR and real-time PCR. (DOC) [file pgen.1002043.s013.doc]

**Supplementary Table 5** Primers for analyzing expression of annotated genes in the refined interval using RT-PCR and real-time PCR.

| Gene | Forward primer (5’-3’) | Reverse primer (5’-3’) | Amplicon (bp) | Tm (^o^C) |
| --- | --- | --- | --- | --- |
| *ANKS1A* | GAATGGCCATAAGGATGTGG | CGCGGTTGTCTTTTATGTTG | 572 | 63 |
| *DEF6* | ATGCTGCTGAGCACCTTCTT | ACATCCTGGACAAGGTGGAG | 235 | 58 |
| *FANCE* | AGACCTCGGAGTTGCTTCTG | GTCCTTGCTGATGGCAGTTT | 281 | 60 |
| *PPARD* | CATGTCTCACAACGCCATTC | CGATGTCGTGGATCACAAAG | 239 | 65 |
| *SCUBE3* | GTTCCAGGGTGAGCCGAGTG | CGGGAAGACGTGCATCGAGA | 963 | 50 |
| *SNRPC* | CTGCAGTGGTAGGAAGCACA | CATACCAGGACGAGGAGGAC | 202 | 65 |
| *TAF11* | AAGGCGAGCTCAAGAGTCAG | GCTTTGAGTTGGGAATCTGC | 450 | 65 |
| *TCP11* | CATACTCGGCTGCTTGTTGA | GTCTGGAAGGCAAGGTCAAG | 191 | 65 |
| *ZNF76* | CGCTGGTAGGGAGTCAGAAG | TGCAGTGTAGTGTGGTGGTG | 351 | 65 |
| *UHRF1BP1* | GCAGCTCCAGGGCTATAGTG | ATCACAAATGTGCAGGTCCA | 574 | 65 |
| *β-actin* ^a^ | TCGATCATGAAGTGCGACGTG | GTGATCTCCTTCTGCATCCTGTC | 113 | 60 |
| *PPARD* ^a^ | CAGCGCCTACCTGAAAAACTTC | GCCTTGCCGGTGAGGAT | 66 | 60 |

^a^ Real time PCR primers; The TaqMan probe for *β-actin* is FAM-ATCAGGAAGGACCTCTACGCCAACACGG-TAMRA, and *PPARD* TaqMan probe is FAM-ACATGACCAAAAAGAAGGCCCGCG-TAMRA.
