# Supplementary material for: A Missense Mutation in PPARD Causes a Major QTL Effect on Ear Size in Pigs
Source: PLoS Genet. 2011 May 5;7(5):e1002043. doi: 10.1371/journal.pgen.1002043 (PMC3088719; doi:10.1371/journal.pgen.1002043)
Supplement: Table S6 — Primers for identification of polymorphisms in the coding and genomic regions of porcine PPARD. (DOC) [file pgen.1002043.s014.doc]

**Supplementary Table 6 Primers for identification of polymorphisms in the coding and genomic regions of porcine *PPARD*.**

| Region | Forward primers (5’-3’) | | Reverse primer (5’-3’) | | Tm (^o^C) ^a^ | | Amplicon (bp) | | SNP ^b^ | |
| --- | --- | --- | --- | --- | --- | --- | --- | --- | --- | --- |
| CDS |  | |  | |  | |  | |  | |
|  | CAGCCAAGTCAGCGTCGTGT | | AGAATGATGGCTGCGATGAA | | 58 | | 1267 | | c.196G>A | |
|  | GCTGGGCTGACGGCAAACGA | | GAGGAGGCAGGGCTATAAGG | | 58 | | 1023 | | c.1238T>G | |
|  | TCGACCACCTCTTCCTCAAC | | CTTGAGGCATGCTCCTCTTC | | 61 | | 1263 | |  | |
|  | TCAGCCTCGCTTTCTCTCTC | | CTGCTGAGGTGAGTGCTGTC | | 64 | | 1127 | |  | |
| Genomic |  | |  | |  | |  | |  | |
|  | GACCAGCCAGAGAACTCAGC | | AAAGGCAGAATGAAGCCTGA | | 53 | | 731 | | g.93G>A, g.276T>C | |
|  | CTGGTAGGCACGAAGATGG | | TTTGAAGGTCTATGAATCCC | | 60 | | 1035 | | g.304C>T | |
|  | | AACTAATGCAGCCCCACAAC | | CGTTATCCTGCCTTTGTCGT | | 62 | | 1146 | | g.2470G>A |
|  | | TGAAACAAGCGACAAAGCAC | | AGGATCAGCAAAGGCAGTGT | | 65 | | 1453 | | g.6429del.G |
|  | | AGTCGCTGTCCCATAACACC | | ACACCACTGACGGTGTTTCA | | 62 | | 753 | | g.10836C>T, g.11334G>A |
|  | | ACAGCGGGTTAAGGTTCCA | | GAGCCTCACATTACTTTCGTC | | 60 | | 1001 | | g.12494A>G |
|  | | CGGATGAACCCTGTAGTCGT | | GGAAAGCTCACCTGCTGTTC | | 63 | | 1021 | | g.12779C>T, g.13149C>T |
|  | | GGAGCTAAGCACAGCCATTC | | AGAGATGCTCACCAGGCACT | | 63 | | 939 | | g.17149A>G |
|  | | AACAAGGCAAGGATGTCCAC | | CATTGTGGCACATGGAGTTC | | 65 | | 1386 | | g.23809A>T |
|  | | AACAAGGCAAGGATGTCCAC | | GTTGTGGGTCCCAATACCAC | | 65 | | 1079 | | g.24246A>G |
|  | | AGAACCGGAAAGGTCCAACT | | GTGCTGAGATCCAAGAAGGC | | 62 | | 932 | | g.28127C>G |
|  | | TCTTTCTCCATGTCCCCTTG | | CTGCTTCTCTCACCCTGTCC | | 62 | | 1251 | | g.31889T>C |
|  | | CCAGTAGCATCCCAAGGTGT | | CTCTCTCCTCTGCCATCCAC | | 65 | | 1368 | | g.32326G>A |
|  | | GCCTGTCCAATCCATCCTT | | GACCATGCTTGTCCCTCCC | | TD | | 1119 | | g.37980A>G |
|  | | GATCGAACCCACCACAACC | | TGACAGAGGCAGGACCAGAG | | TD | | 886 | | g.43563G>A |
|  | | CCCTGAGCGACTATTTCCT | | GGCTATTCTGGGTCTTCTGT | | TD | | 875 | | g.50395G>A |
|  | | CCCTCACCTTCCAATTCTCA | | GTTCTGGAGGAGGCAGTCAG | | 58 | | 809 | | g.51393C>A |
|  | | GATACACTGCGGTAAGACGG | | GAATGTGAACGCCCTAAAGA | | TD | | 1165 | | g.55260T>C |
|  | | ACCGAAGTTTATGCCACAGG | | TTTTGACGTCCTGCTGTCTG | | 66 | | 935 | | g.56969C>T |
|  | | CGCCTGAGTTGTGTGTGAGT | | GCCTGAGTGCTGGTGGTTAT | | 62 | | 995 | | g.61399 G>A, g.61489C>G |
|  | | AACACGGGCTTAATGACAGG | | ATGCACACAGACTGCACACA | | 63 | | 902 | | g.68304T>C, g.68829C>G |
|  | | GGCTCTTCCTGAGGTCTGTGT | | ATCCTGTCCTGCCTGTCTTCT | | 60 | | 528 | | g.69558T>C |
|  | | GGCTGTCGGAATTAAACACAA | | GAGAAGACAGGCAGGACAGG | | 63 | | 947 | | g.70478G>C |
|  | | TAGGCGCTGTAGAGGTGCTT | | TGAGGTAGCCTGCCTGAAGT | | 63 | | 950 | | g.71488G>A |
|  | | GAGAAAGCGAGGCTGAGAGA | | AACAGGTCCAGAGAGGCTGA | | 64 | | 773 | | g.72885T>G |

^a^ TD, touchdown PCR profile with 13 cycles at an annealing temperature of 72 ^o^C (-1 ^o^C/cycle) and 28 cycles at an annealing temperature of 55 ^o^C.

^b^ SNP markers were genotyped using the ABI SNpshot protocol for analysis of haplotype phylogenies and linkage disequilibrium in the *PPARD* region.
