# Supplementary material for: A Missense Mutation in PPARD Causes a Major QTL Effect on Ear Size in Pigs
Source: PLoS Genet. 2011 May 5;7(5):e1002043. doi: 10.1371/journal.pgen.1002043 (PMC3088719; doi:10.1371/journal.pgen.1002043)
Supplement: Table S7 — Primers for real time RT-PCR analysis in cultured cells. (DOC) [file pgen.1002043.s015.doc]

**Supplementary Table 7** Primers for real time RT-PCR analysis in cultured cells ^a^

| Gene | Forward primer (5’-3’) | Reverse primer (5’-3’) | Amplicon (bp) |
| --- | --- | --- | --- |
| *β-catenin* | GCAGCGACTAAGCAGGAAG | GATGACGAAGAGCACAGATGG | 241 |
| *GAPDH* | GGTGCTGAGTATGTCGTGGAG | GTCTTCTGGGTGGCAGTGAT | 290 |
| *c-MYC* | ATTGATGTGGTGTCTGTGGAG | GTAGTTGTGCTGGTGAGTGG | 144 |
| *PPARD* | CATCAGGCTTCCACTACGG | CACTTGTTGCGGTTCTTCTTC | 133 |
| *Sox9* | TGAACGAGAGCGAGAAGAGAC | GGCGGACCCTGAGATTGC | 144 |

^a^ qPCR profiles are 95 ^o^C for 15 s, 1 cycle; 40 cycles of 95 ^o^C for 5 s and 60 ^o^C for 30 s.
